# Statistical Approach of Gene Set Analysis with Quantitative Trait Loci for Crop Gene Expression Studies

**DOI:** 10.3390/e23080945

**Published:** 2021-07-23

**Authors:** Samarendra Das, Shesh N. Rai

**Affiliations:** 1 Division of Statistical Genetics, ICAR-Indian Agricultural Statistics Research Institute, PUSA, New Delhi 110012, India; samarendra.das@louisville.edu; 2 Biostatistics and Bioinformatics Facility, JG Brown Cancer Center, University of Louisville, Louisville, KY 40202, USA; 3 School of Interdisciplinary and Graduate Studies, University of Louisville, Louisville, KY 40292, USA; 4 Department of Pharmacology and Toxicology, University of Louisville, Louisville, KY 40202, USA; 5 Alcohol Research Center, University of Louisville, Louisville, KY 40202, USA; 6 Hepatobiology and Toxicology Center, University of Louisville, Louisville, KY 40202, USA; 7 Department of Bioinformatics and Biostatistics, University of Louisville, Louisville, KY 40202, USA

**Keywords:** gene set, RNA-seq, gene expression, gene set analysis, quantitative trait loci, false discovery rate

## Abstract

Genome-wide expression study is a powerful genomic technology to quantify expression dynamics of genes in a genome. In gene expression study, gene set analysis has become the first choice to gain insights into the underlying biology of diseases or stresses in plants. It also reduces the complexity of statistical analysis and enhances the explanatory power of the obtained results from the primary downstream differential expression analysis. The gene set analysis approaches are well developed in microarrays and RNA-seq gene expression data analysis. These approaches mainly focus on analyzing the gene sets with gene ontology or pathway annotation data. However, in plant biology, such methods may not establish any formal relationship between the genotypes and the phenotypes, as most of the traits are quantitative and controlled by polygenes. The existing Quantitative Trait Loci (QTL)-based gene set analysis approaches only focus on the over-representation analysis of the selected genes while ignoring their associated gene scores. Therefore, we developed an innovative statistical approach, GSQSeq, to analyze the gene sets with trait enriched QTL data. This approach considers the associated differential expression scores of genes while analyzing the gene sets. The performance of the developed method was tested on five different crop gene expression datasets obtained from real crop gene expression studies. Our analytical results indicated that the trait-specific analysis of gene sets was more robust and successful through the proposed approach than existing techniques. Further, the developed method provides a valuable platform for integrating the gene expression data with QTL data.

## 1. Background

Gene expression (GE) studies including RNA sequencing (RNA-seq) and microarrays are powerful techniques for studying expression dynamics and regulation of genes in human and non-human genomes. RNA-seq has surpassed microarrays by providing better quantification of the expression of genes with higher accuracy and better reproducibility [1]. Through RNA-seq, expression levels of genes are measured in terms of discrete read counts obtained by mapping the sequence reads to the reference genome followed by quantification of transcript abundance [2]. It also allows studying alternative splicing [3], new coding and non-coding RNA transcripts, and long non-coding RNAs [4,5]. In other words, RNA-seq is much more prevalent and efficient, as it answers a much more comprehensive range of questions than the existing microarrays technology. Further, differential expression (DE) analysis is one of the significant downstream analyses performed on the RNA-seq count data to detect DE genes with higher resolution than microarrays across the two different experimental conditions [6]. Biologists considered this DE analysis as the end of their analysis. In order to interpret the long list of DE genes in the context of the underlying phenotypic differences and gain insights into biological mechanisms [7], secondary genomic data analytics, e.g., gene set analysis (GSA), are usually widespread. GSA allows us to interpret the high-throughput RNA-seq count data in a broader biological context.

GSA methods were initially developed for microarrays but later extended to RNA-seq [7]. In GSA, the preparation of a ranked gene list (i.e., DE analysis) is a major process that depends on the data’s nature and distributional properties. For instance, GSA approaches for microarrays deal with continuous data and are expected to follow Gaussian distribution. Contrarily, GE data in RNA-seq are non-negative counts (discrete in nature) and assumed to follow a negative binomial distribution. Therefore, it may be improper to use GSA techniques meant for microarray data directly with RNA-seq data. Initially, GSA for RNA-seq data analysis was adopted from microarrays with data transformation; subsequently, new approaches exclusively for RNA-seq were also developed [8]. For instance, the VOOM-normalization technique was used to normalize the read counts for sequence-depths, then microarray GSA approaches are applied to the normalized data [9]. Then, specialized GSA methods for RNA-seq were developed, which includes GOseq [10]. It performs over-representation of gene ontology (GO) categories enriched with the long list of DE genes in RNA-seq data. Further, an easy-to-use web application, iDEP (Integrated Differential Expression and Pathway analysis), was developed for the in-depth analysis of RNA-seq data based on the available pathway information [11]. Both the methods belong to the over representation analysis (ORA) category of the GSA, which uses the GO and pathway information to analyze the RNA-seq data [10,11]. These GSA methods only consider the number of DE genes alone and ignore any values associated with them, such as read counts, DE scores, etc. By discarding this information, ORA-based GSA methods treat each gene equally by assuming that each gene is independent of the others, which is quite unrealistic in biology [12]. The ORA typically focuses on the genes in the gene set and discards the others [13]. Hence,, GSA methods based on gene enrichment statistic(s), such as AbsFilterGSEA [14,15], seqGSEA [16], ssGSEA [17], EGSEA [18], GSVA [19], GSEPD [20], and RNA-Enrich [21], were developed exclusively for RNA-seq data analysis. Further, the researchers can find the details of these methods’ reviews, their comparison, and their unique features in recent literature [7,13]. However, these techniques also suffer from limitations, such as using the DE score to prepare ranked transcripts/gene list but further ignore this information for gene set testing. These approaches also use the data transformation technique, through which over-dispersion, zero inflation, count nature, and other inherent nature of RNA-seq data are lost [13].

The contemporary GSA approaches mostly use GO and pathway information for analyzing gene sets [10,11,14,15,16,17,18,19,20,21] and are very useful in establishing the links of gene sets with underlying biological/molecular processes. However, in plant and complex disease biology, such approaches may not show any formal relationship between the underlying genotypes and the trait/phenotype. This is because most of the traits are quantitative in nature and controlled by polygenes [22,23,24,25]. Apart from the GO and pathways, other biological information, such as Quantitative Trait Loci (QTL), are available in public domain databases that can be effectively used in GSA to gain biological insights into the etiology of complex diseases in humans as well as other non-human organisms (e.g., plants). For this purpose, statistical approaches and tools were developed to perform GSA with genetically enriched QTL data for GE microarray studies [25]. These approaches may have immense use for performing trait/QTL enrichment analysis of gene sets. For instance, the QTL-enriched gene sets can be used for molecular breeding programs for biotic/abiotic stress engineering in plants. 

A Gene Set Validation with QTL (GSVQ), or microarray-QTL, test was developed based on the enrichment testing of selected gene sets with the QTL regions through hypergeometric statistical tests [26]. This approach is not so statistically sound, as it violates the basic assumptions of the hypergeometric statistical tests (e.g., sampling without replacement) [25]. To tackle these issues in analyzing the gene sets with QTL, another approach, i.e., Gene Set Analysis with QTL (GSAQ) for microarrays, has been reported in the literature and found to be better and robust compared to GSVQ [25]. However, GSAQ has some serious limitations. First, it only considers the genes present in the selected gene set but failed to use the corresponding DE scores of genes present in that gene set. Second, GSAQ treats each gene as equally important by assuming the genes are independently and identically distributed, contrary to fundamental biology. Third, GSAQ and GSVQ approaches use only the most significant genes while discarding other genes. For instance, a gene input list from microarrays is obtained by setting the arbitrary threshold(s) for fold-change and *p*-values as 1.5 and 0.01, respectively. With this method, marginally less significant genes (e.g., fold-change~1.499 and *p*-value~0.011) are missed, resulting in information loss for some essential genes. Under these circumstances, the statistical methodologies for GSA with QTL requires further improvements and advances, which will be very helpful in unraveling genotype–phenotype relationships in plants or in complex diseases.

We, therefore, propose a novel statistical approach, i.e., GSQSeq, for analyzing gene sets with trait-enriched QTL data for gene expression studies, including RNA-seq. This approach considers the genes present in the gene set and their corresponding DE scores to analyze the gene set in the presence of the trait-specific QTL data. Here, the gene sets’ enrichment significance was assessed through the *p*-values computed using the developed test statistic(s). Further, we evaluated the proposed method’s performance with respect to the existing approaches, including GSVQ and GSAQ, through performance metrics such as False Discovery Rate (FDR) and −log_10_(*p*-value) on multiple real crop datasets. For this purpose, we used five GE datasets obtained from microarrays and RNA-seq studies in rice. Our analytical findings indicate that the developed approach more successfully detected the QTL-enriched gene sets than the existing techniques. Additionally, GSQSeq has a robust performance over the existing traditional methods, including GSVQ and GSAQ, when assessed on multiple rice gene expression datasets. We implemented the proposed statistical approach in a freely available R software package for the benefit of users.

## 2. Material and Methods

### 2.1. Real Microarray Datasets

Rice GE experimental datasets were collected from the Gene Expression Omnibus (GEO) database of NCBI for Affymetrix platforms GPL2025 (www.ncbi.nlm.nih.gov/geo/query/acc.cgi?acc=GPL2025, accessed on 20 December 2020) [27]. We used rice GE datasets; as it is a model crop plant, a massive amount of GE and QTL datasets are publicly available, and its genome is well annotated. Here, we used the rice GE datasets for the GPL2025, as this platform contains as many as 220 microarray experiments (series) comprising 3480 samples/subjects of *Oryza sativa* L. compared to other platforms. Among these 3480 samples, 150 experimental samples related to four different biotic and abiotic stresses for rice were taken in this study. Initially, we collected the raw CEL file data for the cold, drought, fungal, and insect stresses from 4, 5, 2, and 1 microarray studies, respectively, in rice.

### 2.2. Pre-Processing of Rice Microarrays Datasets

The raw CEL files of the collected samples were processed using the Robust Multichip Average (RMA) algorithm available in the *affy* Bioconductor package of R [28]. This procedure involves background correction, quantile normalization, and summarization by the median polish approach [29]. Further, the log2 scale transformed expression data from RMA for the collected experimental samples were used for meta-analysis of each stress separately to remove the outlier samples (Supplementary Document S1). In other words, microarray GE samples for cold, drought, insect, and fungal stresses were integrated through meta-analysis (under the parameter settings in Supplementary Table S2) to obtain the meta-data. For example, the drought stress dataset, originating from 5 independent studies available in the GEO database under the accession numbers GSE6901, GSE26280, GSE21651, GSE23211, and GSE24048, were integrated through meta-analysis. Now, the meta-data consists of expression measurements of 57,381 genes for over 70 samples (case: 35 and control: 35). Then, these meta-datasets for the respective stresses were further used to remove the control and irrelevant features through the preliminary gene selection. This process reduces the computational complexity and data sets’ dimensions. For instance, out of 57,381 genes in the drought stress, the control (123) and irrelevant (48,180) genes were filtered out by setting the fold change and *p*-value (from *t*-test) parameters as 1 and 0.05, respectively, through the preliminary gene selection. The summary and details of these datasets are given in Table 1 and Supplementary Table S1, respectively. The detailed descriptions of data collection, pre-processing, meta-analysis, and preliminary gene selection of these datasets are given in Supplementary Document S1. The QTL datasets for the stresses in rice, *viz.* drought, cold, insect, and fungal, were collected from the Gramene QTL database (http://www.gramene.org/qtl/, accessed on 15 December 2020) [30]. The positions of the QTLs for each stress were mapped to a reference genome through the MSU rice genome browser [31]. The lists of the respective stress-responsive QTLs and their mapped positions on the rice genome are given in Supplementary Document S4.

### 2.3. Real RNA-Seq Dataset

The raw sequence datasets of rice (Japonica group) under salinity stress were collected from the NCBI database (https://www.ncbi.nlm.nih.gov/, accessed on 18 December 2020). The datasets were generated from Illumina HiSeq 2000 sequencing platform and available in the GEO database with platform GPL13834 (www.ncbi.nlm.nih.gov/geo/query/acc.cgi?acc=GPL13834, accessed on 18 December 2020). This platform consists of 323 samples and 29 series of *Oryza sativa*. Among these datasets, we used the sequence data pertaining to GSE109341 accession, submitted by Formentin et al. on 18 January 2018, and last updated on 13 June 2018, to test our proposed approach [32]. Unlike other datasets, GSE109341 has a relatively large number of samples belonging to two contrasting conditions, i.e., treated vs. control. Further, the sequence datasets were generated from root and leaf tissue samples under untreated and treated plants of Vialone nano and Baldo rice genotypes. Each sample was made of 6 pooled plants with three biological replicates. The raw data, in .fastq files, of these samples were selected from the Sequence Read Archive (SRA) database (https://www.ncbi.nlm.nih.gov/sra/, accessed on 18 December 2020) for further statistical analysis. The detail description about the raw data and their pre-processing is given in Supplementary Document S2. The salinity stress QTL dataset was collected from the Gramene QTL database (http://www.gramene.org/qtl/, accessed on 15 December 2020) [30]. The salinity responsive QTLs’ positions are mapped to the rice genome through the MSU rice genome browser [31] and are given in Supplementary Document S4.

### 2.4. RNA-Seq Preprocessing and Read Alignment

The single-end Illumina raw sequence reads were downloaded from the SRA database using the SRA toolkit (version 2.9.1-1). The raw reads were then preprocessed with the Trimmomatic toolkit (version 0.38), which involves removing adapter sequences, quality filtering, etc. Further, the overall quality of the preprocessed results was manually inspected using quality reports generated by FastQC (version 0.11.7). Then, the preprocessed reads were mapped with HISAT (hisat2-2.1.0) [33] on the *Oryza sativa* v. Nipponbare reference genome, downloaded from the MSU Rice Genome Annotation Project version 7.0 (http://rice.plantbiology.msu.edu/, accessed on 18 December 2020) [31]). The mapping of sequence reads to the reference genome allows identifying their genomic positions. The gene coordinates file (.GFF3) was collected from the MSU rice genome browser, which helps to map the reads to spanning splice junctions to get the genes’ chromosomal positions.

### 2.5. Transcript Assembly and Quantification

The success of the RNA-seq data analysis requires accurate reconstructions and proper quantification of all the isoforms expressed from each gene [2]. Here, we used and executed the StringTie tool (version 1.3.4d) [34] to assemble transcripts from the RNA-seq reads aligned to the genome, which primarily involves two steps. First, grouping the reads into distinct gene loci and then assembling each locus into as many isoforms. After assembling the transcripts with StringTie, we used the *gffcompare* tool [35] to assess the success of matching the assembled transcript with pre-annotated genes, either fully or partially. It was also used to identify the novel transcripts discovered in the mapping process. 

The given experiment involved multiple RNA-seq samples generated for two varieties (with two tissue samples) under two different contrasting conditions (salinity treated vs. untreated). Hence, genes and transcripts present in one sample are rarely identical to others due to varied sequencing depth. So, they need to be assembled in a consistent manner for which the mapping results for individual samples can be compared. For this purpose, we executed the merge function implemented in the StringTie tool, which prepares a final list of genes by merging all the genes found in any of the samples.

### 2.6. Notations

Let, $Y_{ij}$: read counts of *i*th (*i* = 1, 2, …, *N*) gene in *j*th (*j* = 1, 2, …, *M*) sample/library; **Ω**: collection of all genes present in the RNA-seq data (i.e., whole gene list); ***G****:* gene set selected from **Ω**; *N:* size of **Ω**; *M*: number of samples/libraries; *n*: size of ***G***; $\mu_{ij}$: mean of *i*th gene in *j*th sample/library; $\theta_{ij}(=\varphi_{ij}^{-1}$) and $\varphi_{ij}$: size and dispersion parameters respectively of *i*th gene in *j*th sample/library; ***Q***: set of associated QTLs; *D_i_*: differential gene expression score for *i*-th gene; and *T_i_* be the threshold placed at the *i-th position* in gene ranked list, which divides the gene list into *G* and *G^c^* = (Ω−G).

### 2.7. Preparation of Gene Ranked List

We used the edgeR R package [36,37] to prepare the gene ranked list for the RNA-seq read count data. The edgeR tool models the $Y_{ij}$ through a negative binomial model, and its Probability Mass Function (PMF) is given in Equation (1). The expressions of the expected value and variance of the observed read counts ($Y_{ij}$) are given in Equations (2) and (3), respectively. For each gene, the expected value ($\mu_{ij}$) is assumed to be the product of the total number of reads (i.e., library size) and the (unknown) relative abundance ($Z_{ij}$) of that gene in the current experimental condition, expressed in Equation (4). Here, $V\left(Y_{ij}\right)$ is a function of $\mu_{ij}$, as shown in Equation (3), and which requires the estimation of the over-dispersion parameter ($\varphi_{ij}$). So, edgeR estimates $\varphi_{ij}$ using a conditional Maximum Likelihood Estimation procedure, conditioning on the total read count for each gene, and an empirical Bayes procedure to shrink the dispersions toward a consensus value [36]. Here, we used the Likelihood Ratio Test (LRT) statistic(s) to calculate the DE scores of genes under a generalized linear model framework, given in Equation (5). We computed the DE scores of the genes through executing *glmLRT* implemented in edgeR R package [37], and finally prepared the gene ranked list. Further, we used the *t*-test statistic(s) to compute the DE scores of genes for preparing the gene ranked list for microarray GE data. The detailed procedure is given in Supplementary Document S3.

The PMF of the Negative Binomial distribution is expressed as:

(1) $$f_{NB}\left(y\right)=P\left[Y_{ij}=y\right]=\frac{G\left(y+\theta_{ij}\right)}{G\left(y+1\right)G\left(\theta_{ij}\right)}{\left(\frac{\theta_{ij}}{\theta_{ij}+\mu_{ij}}\right)}^{\theta_{ij}}{\left(\frac{\mu_{ij}}{\theta_{ij}+\mu_{ij}}\right)}^{y}\;\;\;\;\;\;\;\;\;\;\forall \;y=0,\;1,\;2,\;\ldots$$

where $\mu_{ij}\geq 0;\;\theta_{ij}>0$ are the parameters of NB distribution, and *G*(.): gamma function. Then, the expected value and variance of $Y_{ij}$ is shown as:

(2) $$E\left(Y_{ij}\right)=\mu_{ij}$$

(3) $$V\left(Y_{ij}\right)=\mu_{ij}+\frac{\mu_{ij}{}^{2}}{\theta_{ij}}=\mu_{ij}+\varphi_{ij}$$

If $\varphi_{ij}\to 0\;\left(No\;dispersion\right)\dot{\Rightarrow }NB\left(\mu_{ij},\theta_{ij}\right)\;\to Poisson\left(\mu_{ij}\right)$

(4) $$\mu_{ij}=s_{j}E\left(Z_{ij}\right)$$

(5) $${log}_{2}E\left(Z_{ij}\right)=\beta_{0i}+\beta_{1i}X_{j}$$

where $s_{j}$: size factor of *j*th library/sample; $Z_{ij}$ represents the true (unknown) concentration of reads for *i*th gene of *j*th library/sample; $X_{j}$ is simply the binary indicator of the group membership of *j*th library/sample (case: 1 and control: 2); $\beta_{0i}$: logarithm of mean parameter for the *i*th gene in the reference control group; and $\beta_{1i}$: log fold-change parameter for the *i*th gene.

### 2.8. Proposed GSQSeq Approach

Earlier developed GSVQ and GSAQ approaches were based on the ORA of the QTL hit genes (i.e., genes overlapped with QTL regions) in the selected gene set through hypergeometric distribution [25,26]. This approach only considered the genes in the selected gene set but ignored their corresponding DE scores. Hence, we developed the GSQSeq approach that can integrate the available DE scores of the selected genes with QTL analysis of the gene sets. For this purpose, we developed a scoring function for the gene set in GSQSeq that combines features from over-representation and shifted expression-based approaches [38]. Here, the scoring function is computed using hypergeometric distribution based on enrichment scores weighted with the DE scores computed through tests such as *t*-test, fold change, etc. Alternatively, GSQSeq uses the long list of genes (which should be ordered based on the DE scores) along with the corresponding vector of the DE scores. It divides the input gene list into the selected gene set (G) and not-selected gene set (*G^c^*) based on the chosen threshold value. Then, it calculates the test statistic, given in Equation (6), for every gene set of the ordered gene list taken at each threshold value [39] by using the following procedure.

The GSQSeq uses a function to calculate the difference between the sum of differential gene expression test scores for *G* and *G^c^* and is expressed in Equation (6).

(6) $$SD_{GQ}=\underset{\begin{array}{c} i\in G\\ i\in Q \end{array}}{\sum }D_{i}-\underset{\begin{array}{c} i\in G^{c}\\ i\in Q \end{array}}{\sum }D_{i}$$

This calculation is repeated for each threshold value, *T_i_*. It is worthy to note that the *T_i_*s are chosen based on the user’s discretion under the constraint that $T_{i}\in \left\{\min\left(D_{i}\right),\max\left(D_{i}\right)\right\}$.

Therefore, to perform the gene set analysis with the underlying trait-specific QTLs for GE studies, including RNA-seq data, we developed the GSQSeq approach under a sound computing framework. In other words, it can be used to evaluate the statistical significance of selected gene sets related to a specific trait based on available QTL information. Under the GSQSeq approach, the following hypotheses can be constructed for testing purposes.

**H_0_**: *Genes in G are at most as often overlapped with the QTL regions as the genes in G^c^ (i.e.,* $SD_{GQ}=0$ *).*

**H_1_**: *Genes in G are more often overlapped with the QTL regions as compared to genes in G^c^ (i.e.,* $SD_{GQ}>0$ *).*

The above constructed null hypothesis is a competitive hypothesis as it considers the genes from both *G* and *G^c^* [13,40,41]. Here, the *H_0_* tells that the QTL hit gene set members and non-members are distributed randomly across the gene list. Now, the QTL hits of the genes present in *G* can be determined through the indicator function given in Equation (7).

(7) $$I_{q}\left(g_{i}\right)=\{\begin{array}{c} 1\;\;\;\;\;\;\;if\;g_{i}{}^{c}\left[a,\;b\right]\in q^{c}\left[d,\;e\right]\\ 0\;\;\;\;\;\;if\;g_{i}\left[a,\;b\right]\notin q^{c}\left[d,\;e\right] \end{array}$$

where $g_{i}\in G$; *a* and *b* represent the start and stop positions (in terms of base pairs) in chromosome *c* of the gene $g_{i}$; q∈Q; and *d* and *e* represent the start and stop positions (in base pairs) in chromosome *c* of the QTL *q.*

The *NQHits* statistic [25], based on the overlapping of the selected genes with the QTL regions (given in Equation (7)), is shown in Equation (8).

(8) $$NQHits=\sum_{i=1}^{n}\sum_{q}^{\left|Q\right|}I_{q}\left(g_{i}\right)$$

It is important to note that the existing techniques, including GSVQ and GSAQ, use the *NQHits* test statistic given in Equation (8) for gene set testing. This test statistic considers each gene as equally important and does not consider their DE scores. Hence, we developed the $SD_{GQ}$ test statistic (Equation (6)). However, the $SD_{GQ}$ alone cannot be used for enrichment testing of gene sets, as it is unstable due to different sizes of the gene sets G and *G^c^*. Therefore, we used the Z-score transformation of the test statistic ($SD_{GQ}$) (Equation (8)) and which is expressed in Equation (9).

(9) $$Z=\frac{SD_{GQ}-E\left(SD_{GQ}\right)}{\sqrt{V\left(SD_{GQ}\right)}}$$

where $E\left(SD_{GQ}\right)$ and $V\left(SD_{GQ}\right)$ are the expected value and variance of the $SD_{GQ}$, respectively.

Further, we obtained the distribution of the test statistic $SD_{GQ}$, given in Equation (6), under *H_0_*. The expressions for mean and variance of the test statistic are given in Equations (10) and (11), respectively.

(10) $$E\left(SD_{GQ}\right)=2E\left(X\right)E\left(N_{GQ}\right)-nE\left(X\right)$$

(11) $$V\left(SD_{GQ}\right)=4(\frac{V\left(X\right)}{n-1}(E\left(N_{GQ}\right)(n-E(N_{GQ}))-V\left(N_{GQ}\right)+E{\left(X\right)}^{2}V\left(N_{GQ}\right))$$

where *X*: differential gene expression test scores of the genes in the gene set; $N_{GQ}$: number of gene set members in *G* got QTL hits; *E*(.): expected value; and V(.): variance.

The estimate of the expected value of $SD_{GQ}$ (Equation (10)) is a linear function of the expected value of the hypergeometric distribution of $N_{GQ}$, the expected value for the differential gene expression test scores in the analyzed selected gene set, *E*(*X*), and the size of the selected gene set, *n*. The E(X) and V(X) in Equations (10) and (11) were computed through the method proposed by Newton et al. (2007) [42], i.e., calculating the mean and variance values of the DE test scores (*X*) for all the genes in the analyzed gene set followed by normalization.

It is worthy to note that the $N_{GQ}$ given in Equations (10) and (11) is same as the *NQHits* statistic proposed by Das et al. (2018) [25] and implemented in the existing GSAQ approach [25], and its expression is shown in Equation (8).

Here, the $N_{GQ}$ follows a hypergeometric distribution and its PMF can be given as:

(12) $$P\left[N_{GQ}=v\right]=\frac{\left(\begin{array}{c} V\\ v \end{array}\right)\left(\begin{array}{c} N-V\\ n-v \end{array}\right)}{\left(\begin{array}{c} N\\ n \end{array}\right)}$$

where *V*: total number of genes covered by the QTLs in the whole **Ω** and *v*: number of genes in ***G*** that are covered by QTLs.

The expected value and variance of the $N_{GQ}$, given in Equation (8), can be expressed in Equations (13) and (14), respectively.

(13) $$E\left(N_{GQ}\right)=\frac{nV}{N}$$

(14) $$V\left(N_{GQ}\right)=\frac{nV\left(N-V\right)\left(N-n\right)}{(N-1)N^{2}}$$

Under *H_0_*, the Z-transformation of the test statistic given in Equation (9) follows a standard normal distribution (under statistical assumptions), i.e., Z ~ N(0,1). Through this property, the statistical significance value for the selected gene set, *G*, was computed. Similarly, this procedure was repeated for all the *K* gene sets obtained by placing the threshold, *T_i_*, (*i* = 1, 2, …, *K*) (K≤N) at *K* different places in the ranked gene list. Then, we adjusted the gene sets’ statistical significance values through the multiple hypothesis testing corrections, and the procedure is given as follows.

Let $p_{1},\;p_{2},\ldots ,\;p_{K}$ be the corresponding *p*-values for all the *K* gene sets, and *α* be the level of significance. Here, we assume that all gene sets are equally important for trait development. Hence, we employed the Hochberg procedure [43] to correct the multiple testing and compute the adjusted (*adj.*) *p*-values for gene sets. It is worthy to note that Hochberg’s procedure is computationally simple, quite popular in genomic data analysis [44], and more powerful than Holm’s method [45]. The algorithm for Hochberg’s procedure [43] is as follows.

Step 1. If $p_{\left(l\right)}>\alpha$, then retain the corresponding null hypothesis ($H_{\left(l\right)}$) and go to the next step. Else, reject it and stop.

Step 2. i=2, 3, …,K−1. If $p_{\left(K-i+1\right)}>\alpha /i$, then retain $H_{\left(K-i+1\right)}$ and go to the next step. Else, reject all remaining hypotheses and stop.

Step 3. *K*. If $p_{\left(1\right)}>\alpha /K$, then retain ($H_{\left(1\right)}$). Else, reject it.

Now, the *adj. p*-values are given recursively beginning with the largest *p*-value [43]:

(15) $$\tilde{p_{\left(i\right)}}=\{\begin{array}{l} p_{\left(i\right)\;\;\;\;\;if\;i=K\;\;\;\;\;\;\;\;\;\;\;\;\;\;\;\;\;\;\;\;\;\;\;\;\;\;\;\;\;\;\;\;\;\;\;\;\;\;\;\;\;\;\;\;\;\;\;}\\ \min(\tilde{p_{\left(i+1\right)}},\;\left(K-i+1\right)p_{\left(i\right)}) \end{array}\;if\;i=K-1,\;\ldots ,1$$

**Figure 1:**
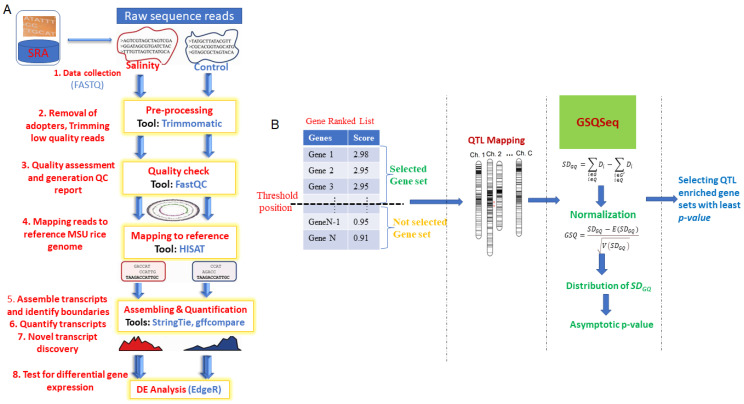
Operational procedure for GSQSeq approach. (**A**) Outlines of RNA-seq data analysis. (**B**) Analytical steps involved in GSQSeq proposed approach. Various steps involved in RNA-seq data analysis are described, starting from data collection to differential expression analysis. The outlines of the analytical steps involved in proposed GSQSeq approach are described. The major steps include: (i) preparation of the gene ranked list after differential expression analysis of RNA-seq count data and selection of gene sets.

Further, based on the computed *adj. p*-value*s*, the underlying QTL enrichment significance of the selected gene sets was assessed. In other words, a lesser value of *adj. p*-value indicates more QTL enrichment of the selected gene set for the target trait development and vice-versa. Similarly, we also computed the False Discovery Rate (FDR) for the selected gene set. The outlines and key analytical steps of the proposed GSQSeq approach are shown in Figure 1.

## 3. Results and Discussion

### 3.1. Mapping Results

From the SRA database of NCBI, a total of 542,309,740 single-end reads (with 50 base pair length) were obtained for 24 libraries. The number of read sequences in each library and the GC content is given in Table 2. The average number of reads per library was found to be 22,596,239, with a CV of 0.169 (=16.9 %). After pre-processing with Trimomatic, the above summary statistic was reduced to 22,353,215 as the mean library size with a CV of 0.171 (=17.1%). However, through pre-processing, the average library size was reduced compared to that of raw sequence datasets, but, the variability among the libraries remained unchanged. Then, the qualities of the read sequences after removal of the adapter sequences were assessed through the FastQC tool, and the results are given in Supplementary Figures S2–S4. From the distribution of the quality scores over the base pairs, it was observed that the quality scores of the sequence reads are above 30, which indicated better qualities of the samples/libraries (Figures S2–S4). In other words, we could not trace any universally low-quality reads in the salinity stress data. Therefore, the processed sequence datasets can be used for further analysis, such as mapping to the rice reference genome followed by quantification transcript abundance. It was observed that most of the pre-processed reads (94.3%) were successfully mapped to the rice reference genome. Out of these mapped reads, 2.87% of reads were mapped to more than one position and subsequently discarded from further analysis. Through StringTie, we quantified the transcriptomic abundance of the transcripts/genes over 24 samples, resulting in the read count data of 55,801 genes. This generated read count data matrix was used to prepare the ranked gene list through the downstream DE analysis.

### 3.2. Genes Ranked List Preparation

#### 3.2.1. Rice RNA-Seq Data

The read sequence count data for each sample/library belonging to two contrasting classes, i.e., salinity treated vs. control, as given in Table 2, were used for the DE analysis. The DE analysis was performed through the edgeR R package (v. 3.30.3) implemented in R software (v. 4.0.1) (Supplementary Document S3). The DE test statistic(s) for the genes were computed through LRT statistic(s). Based on the LRT statistic’s absolute value, the genes were arranged in descending order to prepare the gene ranked list. Different threshold (*T_i_*) values are then placed on the gene ranked list to select different gene sets. Through this process, gene sets of sizes such as 200, 300, 400, …, 2000 are selected from the ranked gene list for further analysis with the underlying salinity responsive QTLs (Supplementary Document S3).

#### 3.2.2. Rice Microarray Datasets

The log2 scale transformed expression data from the RMA for the selected experimental samples (Supplementary Document S1) for the cold, drought, fungal, and insect stresses were used to prepare the gene-ranked list through the DE analysis. Here, the DE analysis was performed through a *t*-test, and the test statistic(s) for the genes were computed from the *t*-test. The genes were arranged in descending order for the preparation of the gene-ranked list. Then, different values of the *T_i_* are placed in various positions on the gene-ranked list to select different gene sets. Through this process, gene sets of sizes such as 200, 300, 400, …, 2000 are selected from the ranked gene lists for each dataset. 

### 3.3. Distribution of the Test Statistic(s)

**Figure 2:**
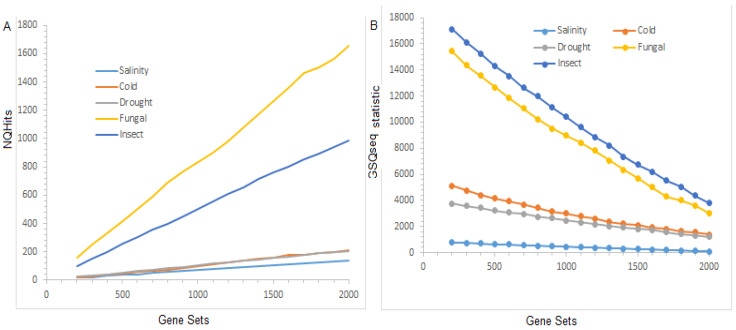
Distribution of test statistic(s) over the gene sets. (**A**) Distribution of *NQHits* test statistic over the selected gene sets. (**B**) Distribution of $SD_{GQ}$ test statistic over the selected gene sets. The distribution of the test statistic(s), such as *NQHits* and $SD_{GQ}$, are shown for salinity stress (RNA-seq data), drought stress, cold stress, fungal stress, and insect stress microarray datasets.

The distribution of the *NQHits* statistic(s), computed through the existing GSAQ approach (under the parameter settings given in Supplementary Table S9) over the selected gene sets for the different stresses are shown in Figure 2A. Further, the distribution of the $SD_{GQ}$ statistic(s) computed from the GSQSeq approach is also shown in Figure 2B. The distribution of the *NQHits* statistic(s) calculated from the GSAQ approach indicated that the values of the *NQHits* statistic(s) were found to be higher for fungal stress, followed by insect stress, as compared to other datasets (Figure 2A). This trend is due to the fact that a higher number (76) of QTLs are reported for this stress, followed by 57 in fungal stress. Alternatively, the *NQHits* statistic is a linear function of the number of genes present in gene sets, the number of QTLs reported for that stress, and the length of the QTL regions (Figure 2A). Similar interpretations can be made for the distribution of the $SD_{GQ}$ statistic(s) (Figure 2B). However, the *NQHits* statistic did not consider the DE scores of the genes present in the selected gene set. Here, it is worthy to note that the $SD_{GQ}$ is a function of the number of genes, their respective DE scores in the gene set, the number of QTLs reported for that stress, and the QTL regions (Figure 2B).

### 3.4. Proposed Approach for Gene Set Analysis with QTLs

The *NQHits* and $SD_{GQ}$ statistic(s) failed to tell the trait-specific enrichment of the gene sets or association of genotype–phenotype relation. Therefore, we proposed the GSQSeq approach to test the gene sets’ trait-specific enrichments with the underlying QTLs. We also explored the ability of the proposed GSQSeq and the existing methods, including GSVQ and GSAQ, to provide biologically meaningful insights (e.g., establishing genotype-trait-specific phenotype associations) using the real high-throughput GE datasets derived from RNA-seq and microarrays. Through all the three tested GSA approaches, we searched significantly associated gene sets enriched with underlying QTLs selected by a particular gene selection method (e.g., *t*-test in microarrays, edgeR in RNA-seq) in each of the datasets. The results of such analysis are shown in Table 3 and Table 4.

For salinity stress RNA-seq data, the magnitude of −log10 (*p*-value*s*) from the GSQSeq was found to be much higher than that of the existing GSVQ and GSAQ approaches (Table 3). This observation indicated that the GSQSeq approach more often rejected *H_0_* (i.e., equal salinity QTL enrichment of both selected and not-selected gene sets) than GSVQ and GSAQ approaches. Therefore, it was found that the salinity trait-specific analysis of gene sets derived from the RNA-seq study was successful through GSQSeq compared to the GSVQ and GSAQ approaches (Table 3). In other words, the GSQSeq approach performed better in terms of detecting the QTL-enriched gene sets compared to the existing methods. In order to cross-validate these findings on the same RNA-seq data related to salinity stress, we computed the FDR for the GSQSeq, GSAQ, and GSVQ approaches for all the gene sets. The results are given in Table 5 and Table 6. It was observed that the computed values of FDR from the proposed GSQSeq approach for all the selected gene sets are far below those of existing GSAQ and GSVQ approaches (Table 5). Therefore, it can be inferred that the proposed GSQSeq approach was more robust than the GSAQ and GSVQ approaches for performing gene set enrichment testing with salinity trait-specific QTLs.

For cold stress data obtained from microarrays, the values of −log10 (*p*-value*s*) from GSQSeq were observed to be higher than those of the existing GSVQ and GSAQ approaches over all the selected gene sets (Table 3). This finding indicated that the GSQSeq approach more often rejected *H*_0_ (i.e., equal cold QTL enrichment of both the selected and not-selected gene sets) than the GSVQ and GSAQ approaches. Further, the FDR values computed through the proposed GSQSeq approach for all the selected gene sets of sizes 200, 300, …, 200 were found to be least followed by the GSAQ compared to the GSVQ approach (Table 5). Similar findings were observed for drought, fungal, and insect stress datasets in rice (Table 4, Table 5 and Table 6). Therefore, it can be concluded that the proposed GSQSeq approach is much better and more robust than GSAQ and GSVQ for performing gene set enrichment testing with the underlying QTLs for the microarray-based GE studies. Furthermore, we found much greater consistency in QTL-specific gene set enrichment analysis across five different stress scenarios, *viz*. salinity, cold, drought, fungal, and insect, by using GSQSeq than the GSVQ and GSAQ approaches (Table 3, Table 4, Table 5 and Table 6).

The proposed GSQSeq approach is an improved way to perform the trait-specific analysis of the gene sets to establish genotype (polygenes)–phenotype (quantitative trait) association testing with the help of genetically rich QTL data. It is more biologically appealing to establish the association of genes (genotype) in the selected gene set with the underlying QTLs (traits/phenotypes). However, in the existing GSVQ and GSAQ approaches, the genes in gene sets are taken as input to hypergeometric distribution for performing trait enrichment analysis. These approaches violated the basic assumptions of the hypergeometric test (i.e., sampling units must be drawn without replacement) and did not consider the DE scores of the gene set’s genes. Thus, the existing approaches are expected to have poor performance in terms of gene set enrichment. Furthermore, the GSQSeq approach was more successful and useful in detecting the trait-specific QTL-enriched gene sets than the existing methods.

The proposed GSQSeq approach allowed us to statistically test the gene set for enrichment with the underlying QTLs (i.e., rejection of the null hypothesis of the random association of selected genes with QTLs). Through this, a *p*-value was assigned to each selected gene set, which is statistically meaningful to genome researchers and experimental biologists (as the value lies between 0 and 1). The gene sets with lower *p*-values are considered as more enriched with the underlying trait-specific QTLs, and vice-versa. It may be noted that the proposed GSQSeq technique is a two-stage approach. First, it deals with selecting gene sets through the downstream DE analysis from the large GE data. Second, it assesses the QTL enrichment significance of gene sets by using a developed parametric testing procedure. This analysis eases the interpretation of a large-scale experimental GE data by identifying trait-specific enriched gene sets. Instead of focusing on the individual QTL hit gene (i.e., genes overlapped with QTL region), researchers can focus on the QTL-enriched gene sets (polygenes), which tend to be more reproducible, and more interpretable (for quantitative traits).

The proposed GSQSeq approach can be considered a valuable tool for performing gene(s) enrichment analysis in a molecular plant breeding context, as most of the plant traits are quantitative and controlled by polygenes. Further, it provides a valuable tool for integrating the GE data from RNA-seq or microarray studies with genetically rich QTL data to identify potential QTL-enriched gene sets or the sets of QTL candidate genes. These QTL-enriched gene sets may provide valuable input or hypotheses to plant breeders to design their breeding experiments.

## 4. R Software Package

To facilitate the use of the proposed gene set analysis with the QTL approach among the researchers, we developed an R software package that includes GSQSeq R package and accompanying documentation with examples. This package is supplied with the manuscript as supplementary material and is also available in https://github.com/sam-uofl/GSQSeq. The inputs and guidelines for the use of the GSQSeq R package are given in Supplementary Document S5. This software can analyze the gene sets for GE datasets derived from expression studies including microarrays and RNA-seq. For microarray GE data, four different gene selection methods, such as *t*-test, F-score, Maximum Relevance and Minimum Redundancy (MRMR), and Support Vector Machine (SVM) techniques [46], are implemented for the selection of relevant gene sets from the high-dimensional GE data. After that, a selected gene set of a particular ‘size’, obtained from microarray GE data, is analyzed with the underlying QTL data. Both these steps can be executed by implementing the *GSQMicro* function in the GSQSeq R package.

In the developed software, two popular and extensively used DE analytical methods, including edgeR and DESeq2, are implemented in the GSQSeq R package to prepare the gene ranked list for RNA-seq count data. Then, the selected genes obtained from the RNA-seq data are analyzed with the underlying QTLs to establish the genotypes with quantitative trait links. These steps are implemented in the *GSQSeq* function of the GSQSeq R package. Moreover, the QTL enrichment significance of the selected gene sets obtained from both the microarrays and RNA-seq can be assessed through the computed *p*-value*s*. However, different user-specific parameter options are provided to the users for their desired analysis. Hence, the GSQSeq software offers opportunities to get the QTL-enriched gene sets of desired ‘sizes’ from the microarray and RNA-seq GE data.

## 5. Conclusions

In the last decade, GSA has become the primary choice of secondary genomic data analysis for explaining the underlying biology for high-throughput GE studies. Most of the bioinformatics studies look for statistically significant gene sets as either biological interpretations of long gene list in GE data or validation of computationally derived results from DE analysis. Traditionally, the gene sets are analyzed based on the available GO or pathway annotation data. However, such research may not establish the links between gene sets (polygenes) with the underlying quantitative traits. Therefore, in this study, we proposed an innovative statistical approach and tool, i.e., GSQSeq, to analyze gene sets with genetically rich trait data, such as QTL. This approach is an improvement over the existing GSVQ and GSAQ methods, as it considers the DE scores of the genes in the gene list in performing GSA with the QTLs. In other words, the proposed approach may be regarded as the second generation of GSA with QTL as it is an improvement over first-generation GSA methods, such as GSVQ and GSAQ (based on ORA). Through this proposed GSQSeq approach, the statistically meaningful and biologically interpretable *p*-values are assigned to each gene set, which profoundly describes the trait enrichment of the gene sets.

The researchers and molecular biologists may focus only on the QTL-enriched gene sets to frame their hypothesis without considering the long list of genes in the high-throughput GE studies. The comparative performance analysis of the proposed approach with respect to the existing methods indicated its better and more robust performance on multiple crop GE datasets. This approach will have immense plant biology and breeding applications for the identification of trait-enriched gene sets (as plant traits are quantitative) for stress response engineering. Further, the developed tool provides a statistically sound computational environment for integrating high-throughput GE data with genetically rich QTL data. This approach can also be extended to analyzing the GE datasets obtained from single-cell RNA-seq GE studies. Here, due to the unavailability of rice single-cell data sets, we cannot test the performance of the GSQSeq approach on such datasets.

Further, the researcher may consider testing the utility of the GSQSeq tool on other plant and non-plant GE studies as a potential future work. In future, statisticians and biologists may focus on developing the next generations of GSA with QTLs by using graph/network theoretic approaches. These new approaches will analyze the high-throughput GE data more efficiently to understand the biological systems better, which will increase the specificity, sensitivity, utility, and relevance of GSA in GE studies.

## Figures and Tables

**Table 1:** Rice gene expression datasets used in the study.

SN.	Descriptions	Type	# Series	Series Id	# Genes	# Samples	Stress
1	Salinity stress	RAN-seq	1	GSE109341	26454	24 (12, 12)	Abiotic
2	Cold stress	Microarray	4	GSE31077, GSE33204, GSE37940, GSE6901	8840	28 (15, 13)	Abiotic
3	Drought stress	Microarray	5	GSE26280, GSE6901, GSE21651, GSE23211, GSE24048	9078	70 (35, 35)	Abiotic
4	Fungal (blast) stress	Microarray	2	GSE41798, GSE7256	7072	26 (13, 13)	Biotic
5	Insect (brown plant hopper) stress	Microarray	1	GSE29967	7241	18 (12, 6)	Biotic

# Series: number of GEO series for each dataset; # Genes: number of genes; # Samples: number of GEO samples; (x, y): number samples for case and control, respectively; number of classes (e.g., 2 in control vs. case genomic study); Type: study type from which gene expression datasets are derived.

**Table 2:** Description about the RNA-seq data for salinity stress response in rice.

Sampleid	SRA id	Genotype	Tissue	Class	Class Label	Total Seq	%GC	Length
GSM2940029	SRR6502085	Vialone Nano	Leaves	Salinity	1	24449975	53	50
GSM2940030	SRR6502086	Vialone Nano	Leaves	Salinity	1	20017598	53	50
GSM2940031	SRR6502087	Vialone Nano	Leaves	Salinity	1	20827821	52	50
GSM2940032	SRR6502088	Vialone Nano	Leaves	Control	0	21716861	53	50
GSM2940033	SRR6502089	Vialone Nano	Leaves	Control	0	20733565	53	50
GSM2940034	SRR6502090	Vialone Nano	Leaves	Control	0	21422404	54	50
GSM2940035	SRR6502091	Vialone Nano	Roots	Salinity	1	27840276	52	50
GSM2940036	SRR6502092	Vialone Nano	Roots	Salinity	1	23879836	53	50
GSM2940037	SRR6502093	Vialone Nano	Roots	Salinity	1	19205417	51	50
GSM2940038	SRR6502094	Vialone Nano	Roots	Control	0	19717555	54	50
GSM2940039	SRR6502095	Vialone Nano	Roots	Control	0	19368950	54	50
GSM2940040	SRR6502096	Vialone Nano	Roots	Control	0	21735612	53	50
GSM2940041	SRR6502097	Baldo	Leaves	Salinity	1	21831739	53	50
GSM2940042	SRR6502098	Baldo	Leaves	Salinity	1	19588041	52	50
GSM2940043	SRR6502099	Baldo	Leaves	Salinity	1	18806965	53	50
GSM2940044	SRR6502100	Baldo	Leaves	Control	0	23497719	54	50
GSM2940045	SRR6502101	Baldo	Leaves	Control	0	20177524	55	50
GSM2940046	SRR6502102	Baldo	Leaves	Control	0	18961877	54	50
GSM2940047	SRR6502103	Baldo	Roots	Salinity	1	19608758	51	50
GSM2940048	SRR6502104	Baldo	Roots	Salinity	1	24485505	51	50
GSM2940049	SRR6502105	Baldo	Roots	Salinity	1	34395309	54	50
GSM2940050	SRR6502106	Baldo	Roots	Control	0	24854382	53	50
GSM2940051	SRR6502107	Baldo	Roots	Control	0	30769580	52	50
GSM2940052	SRR6502108	Baldo	Roots	Control	0	24416471	51	50

Sample id: GEO sample id; SRA: SRA sample id; class labels for samples (1: treated and 0: control); GC content: content of G, C base pairs in read sequences.

**Table 3:** List of the −log_10_(*p*-value*s*) computed from the proposed GSQSeq and existing (GSVQ and GSAQ) approaches for salinity and cold stress datasets.

	Salinity			Cold		
Size	GSVQ	GSAQ	GSQSeq	GSVQ	GSAQ	GSQSeq
200	1.05	18.85	222.71	0.05	1.00	222.46
300	0.67	8.29	212.71	0.01	2.00	223.02
400	0.69	8.02	211.71	0.08	2.00	224.30
500	0.82	12.99	302.23	0.02	2.00	225.83
600	0.62	5.85	270.66	0.02	2.00	226.60
700	0.75	10.74	226.11	0.01	1.30	227.13
800	0.77	13.23	197.34	0.00	1.30	228.46
900	0.89	14.98	168.77	0.00	1.30	229.41
1000	0.73	9.76	159.48	0.01	1.30	230.45
1100	0.85	14.63	138.47	0.02	1.30	231.46
1200	0.71	11.52	132.49	0.03	1.30	302.59
1300	0.91	17.85	112.50	0.02	1.30	249.00
1400	0.85	14.15	105.22	0.02	0.00	236.69
1500	0.72	8.29	101.95	0.03	1.19	232.29
1600	0.83	11.51	89.95	0.04	1.30	219.10
1700	0.71	8.09	87.65	0.02	1.46	213.22
1800	0.82	10.50	77.54	0.01	1.70	196.50
1900	0.93	14.24	68.47	0.01	2.30	203.26
2000	0.81	10.89	67.15	0.00	2.00	180.41

Size: size of the selected gene sets obtained from the gene expression data; −log_10_(*p*-value) are listed in the table; GSQSeq: proposed approach; GSVQ and GSAQ are existing approaches. Performance analysis of the proposed approach was carried out with the existing approaches on salinity stress RNA-seq data and cold stress microarray expression data. Higher the value of −log_10_(*p*-value), better the gene set enrichment with QTL.

**Table 4:** List of the −log_10_(*p*-value*s*) computed from the proposed GSQSeq and existing (GSVQ and GSAQ) approaches for microarray datasets.

	Drought				Fungal			Insect	
Size	GSVQ	GSAQ	GSQSeq	GSVQ	GSAQ	GSQSeq	GSVQ	GSAQ	GSQSeq
200	0.83	11.80	276.94	0.00	0.00	254.48	0.65	6.36	229.17
300	0.64	9.29	252.76	0.31	0.87	220.28	0.94	13.44	234.13
400	0.45	1.86	254.48	0.06	0.00	202.94	0.69	8.45	236.10
500	0.81	11.66	252.28	0.22	0.02	190.28	1.08	18.20	228.86
600	0.79	12.97	252.16	0.23	0.00	181.58	0.92	13.93	228.69
700	0.49	2.75	252.06	0.34	0.62	219.58	1.23	21.99	228.56
800	0.78	11.56	251.98	1.04	10.44	219.50	0.81	12.97	228.47
900	0.70	10.90	251.92	0.98	5.69	219.43	1.09	17.49	228.39
1000	0.93	14.56	251.86	0.14	0.16	219.38	1.09	15.47	228.32
1100	1.01	15.11	251.81	0.00	0.00	219.33	1.43	23.98	228.26
1200	1.08	19.30	251.76	0.00	0.00	219.28	1.62	25.14	228.21
1300	1.00	18.04	293.50	0.04	0.00	219.24	1.43	24.35	228.17
1400	0.93	15.60	276.15	0.19	0.37	219.20	2.28	28.35	228.12
1500	1.01	13.95	252.76	0.34	1.22	219.17	1.99	27.67	228.09
1600	0.69	9.23	254.48	0.94	6.27	219.13	1.50	21.05	228.05
1700	1.01	14.48	220.28	2.29	19.08	219.10	1.62	21.52	228.02
1800	1.09	16.36	202.94	0.14	0.03	219.08	1.04	16.17	227.99
1900	1.10	17.91	190.28	0.00	0.00	219.05	1.19	20.39	227.96
2000	1.04	19.27	181.58	0.01	0.00	219.02	1.08	13.90	227.94

Size: size of the selected gene sets obtained from the gene expression data; −log_10_(*p*-value) are listed in the table; GSQSeq: proposed approach; GSVQ and GSAQ are existing approaches. Performance analysis of the proposed approach was carried out with the existing approaches on drought, fungal and insect stress microarray gene expression datasets. Higher the value of −log_10_(*p*-value), better the gene set enrichment with QTL, and vice-versa.

**Table 5:** List of the FDRs computed from the proposed GSQSeq and existing approaches from salinity RNA-seq and cold stress microarray gene expression datasets.

		Salt			Cold	
Size	GSVQ	GSAQ	GSQSeq	GSVQ	GSAQ	GSQSeq
200	0.324	2.69 × 10^−18^	6.99 × 10^−160^	0.226	0.10	1.58 × 10^−249^
300	0.424	6.14 × 10^−9^	6.50 × 10^−139^	0.254	0.50	2.96 × 10^−237^
400	0.224	9.97 × 10^−9^	5.63 × 10^−133^	0.352	0.68	6.90 × 10^−233^
500	0.124	2.45 × 10^−13^	2.82 × 10^−302^	0.226	0.46	9.96 × 10^−220^
600	0.238	1.40 × 10^−06^	8.31 × 10^−271^	0.226	0.23	7.09 × 10^−214^
700	0.024	2.86 × 10^−11^	2.46 × 10^−226^	0.344	0.01	3.32 × 10^−197^
800	0.024	1.61 × 10^−13^	1.23 × 10^−197^	0.226	0.18	1.78 × 10^−236^
900	0.424	6.67 × 10^−15^	4.08 × 10^−169^	0.241	0.14	2.07 × 10^−236^
1000	0.224	2.37 × 10^−10^	6.99 × 10^−160^	0.223	0.10	2.37 × 10^−236^
1100	0.224	1.12 × 10^−14^	6.50 × 10^−139^	0.223	0.06	2.67 × 10^−236^
1200	0.224	5.87 × 10^−12^	5.63 × 10^−133^	0.223	0.02	4.47 × 10^−303^
1300	0.254	1.35 × 10^−17^	4.98 × 10^−113^	0.223	0.02	1.58 × 10^−249^
1400	0.064	2.25 × 10^−14^	8.88 × 10^−106^	0.223	0.01	2.96 × 10^−237^
1500	0.274	6.14 × 10^−9^	1.53 × 10^−102^	0.223	0.051	6.90 × 10^−233^
1600	0.224	5.87 × 10^−12^	1.43 × 10^−90^	0.241	1	9.96 × 10^−220^
1700	0.224	9.00 × 10^−9^	2.63 × 10^−88^	0.223	1	7.09 × 10^−214^
1800	0.224	4.59 × 10^−11^	3.22 × 10^−78^	0.223	1	3.32 × 10^−197^
1900	0.524	2.19 × 10^−14^	3.60 × 10^−69^	0.223	1	6.10 × 10^−204^
2000	0.124	2.24 × 10^−11^	7.10 × 10^−68^	0.223	1	3.92 × 10^−181^

Size: size of the selected gene sets obtained from the gene expression datasets; False Discovery Rates are listed in the table; GSQSeq: proposed approach; GSVQ and GSAQ are existing approaches. The performance analysis of the proposed approach was carried out with the existing approaches on salinity stress RNA-seq data and cold stress microarray expression data. Lower the FDR values, better the gene set enrichment with QTL, and vice-versa.

**Table 6:** List of the FDRs computed from the proposed GSQSeq and existing approaches from microarray gene expression datasets.

	Drought			Fungal			Insect	
Size	GSAQ	GSQSeq	GSVQ	GSAQ	GSQSeq	GSVQ	GSAQ	GSQSeq
200	2.52 × 10^−12^	1.04 × 10^−276^	0.996	1	6.24 × 10^−221^	0.224	4.33 × 10^−07^	5.1 × 10^−230^
300	6.14 × 10^−10^	2.20 × 10^−253^	0.996	0.42	8.79 × 10^−253^	0.141	4.36 × 10^−14^	3.0 × 10^−225^
400	0.01383	4.47 × 10^−255^	0.996	1.00	1.10 × 10^−252^	0.215	3.77 × 10^−9^	1.8 × 10^−220^
500	3.21 × 10^−12^	6.24 × 10^−221^	0.996	1.00	6.59 × 10^−249^	0.121	1.19 × 10^−18^	1.1 × 10^−215^
600	1.83 × 10^−13^	8.79 × 10^−253^	0.996	1.00	3.95 × 10^−245^	0.141	1.58 × 10^−14^	6.2 × 10^−211^
700	0.00188	1.10 × 10^−252^	0.996	0.65	2.37 × 10^−241^	0.121	3.24 × 10^−22^	3.7 × 10^−206^
800	3.73 × 10^−12^	1.32 × 10^−252^	0.549	3.42 × 10^−10^	1.42 × 10^−237^	0.172	1.19 × 10^−13^	2.2 × 10^−201^
900	1.61 × 10^−11^	1.54 × 10^−252^	0.549	9.63 × 10^−06^	8.54 × 10^−234^	0.121	5.55 × 10^−18^	1.3 × 10^−196^
1000	6.61 × 10^−15^	1.76 × 10^−252^	0.996	1	5.12 × 10^−230^	0.121	4.98 × 10^−16^	7.5 × 10^−192^
1100	2.12 × 10^−15^	1.98E × 10^−252^	0.996	1	3.07 × 10^−226^	0.101	4.01 × 10^−24^	4.4 × 10^−187^
1200	5.11 × 10^−19^	2.20 × 10^−252^	0.996	1	1.84 × 10^−222^	0.101	4.56 × 10^−25^	2.6 × 10^−182^
1300	5.74 × 10^−18^	4.96 × 10^−294^	0.996	1	1.11 × 10^−218^	0.101	2.13 × 10^−24^	1.5 × 10^−177^
1400	7.92 × 10^−16^	1.04 × 10^−276^	0.996	1	6.64 × 10^−215^	0.098	8.54 × 10^−28^	9.0 × 10^−173^
1500	2.14 × 10^−14^	2.20 × 10^−253^	0.996	0.226411	3.98 × 10^−211^	0.098	2.04 × 10^−27^	5.3 × 10^−168^
1600	6.54 × 10^−10^	4.47 × 10^−255^	0.549	3.44 × 10^−06^	2.39 × 10^−207^	0.101	2.13 × 10^−21^	3.1 × 10^−163^
1700	6.94 × 10^−15^	6.24 × 10^−221^	0.097	1.57 × 10^−18^	1.43 × 10^−203^	0.101	8.14 × 10^−22^	1.9 × 10^−158^
1800	1.65 × 10^−16^	1.30 × 10^−203^	0.996	0.150939	8.61 × 10^−200^	0.123	1.06 × 10^−16^	1.1 × 10^−153^
1900	5.89 × 10^−18^	5.57 × 10^−191^	0.996	2.64 × 10^−01^	5.16 × 10^−196^	0.121	8.51 × 10^−21^	6.4 × 10^−149^
2000	5.11 × 10^−19^	2.65 × 10^−182^	0.996	3.77 × 10^−01^	3.10 × 10^−192^	0.121	1.58 × 10^−14^	3.8 × 10^−144^

Size: size of the selected gene sets obtained from the microarray gene expression data; False Discovery Rates are listed in the table; GSQSeq: proposed approach; GSVQ and GSAQ are existing approaches. The performance analysis of the proposed approach was carried out with the existing approaches on drought, fungal and insect stress microarray datasets. The lower the FDR values, the better the gene set enrichment with QTL, and vice-versa.

## Data Availability

All the secondary data used in this study are publicly available in the NCBI GEO database. The developed R package is publicly available to the users at https://github.com/sam-uofl/GSQSeq.

## Supplementary Materials

The following are available online at https://www.mdpi.com/article/10.3390/e23080945/s1. Document S1: Rice RNA-sequencing dataset collection and pre-processing. Document S2. Differential Expression analysis of RNA-seq data. Document S3. Data collection, pre-processing, meta-analysis and preliminary gene selection for rice. Document S4: Stress(es)-specific Quantitative Trait Loci information for rice (*Oryza sativa* L.). Table S9. Number of gene samples and sizes of gene sample for each selected gene set for GSAQ analysis. Document S5: Guidelines and tutorials for GSQSeq R package. Supplementary Material 1. Example datasets for GSQSeq R package. Supplementary Material 2. GSQSeq R package.
