# Supplementary material for: Statistical Approach of Gene Set Analysis with Quantitative Trait Loci for Crop Gene Expression Studies
Source: Entropy (Basel). 2021 Jul 23;23(8):945. doi: 10.3390/e23080945 (PMC8391627; doi:10.3390/e23080945)
Supplement: Supplementary file 1 [file entropy-23-00945-s001.zip › entropy-1275912-supplementary/Supplementary Materials.pdf]

# Supplementary Materials

# of

# Statistical Approach of Gene Set Analysis with Quantitative Trait Loci for Crop Gene Expression Studies

Samarendra Das[1-4],Shesh N Rai[3-8*]

[1]Division of Statistical Genetics, ICAR-Indian Agricultural Statistics Research Institute, PUSA, New Delhi 110012, India

[2]Biostatistics and Bioinformatics Facility, JG Brown Cancer Center, University of Louisville, Louisville, KY 40202, USA

[3]School of Interdisciplinary and Graduate Studies, University of Louisville, Louisville, KY 40292, USA

[4]Department of Pharmacology and Toxicology, University of Louisville, Louisville, KY 40202, USA

[5]Alcohol Research Center, University of Louisville, Louisville, KY 40202, USA

[6]Hepatobiology and Toxicology Center, University of Louisville, Louisville, KY 40202, USA

[7]Department of Bioinformatics and Biostatistics, University of Louisville, Louisville, KY 40202, USA

**Authors' email addresses:**

SD: samarendra.das@louisville.edu

SNR: shesh.rai@louisville.edu

***To whom correspondence should be addressed-** email: shesh.rai@louisville.edu.

**<u>Document S1</u>. Data collection, pre-processing, meta-analysis and preliminary gene selection for rice Microarray datasets.**

***Data selection***

The six different gene expression datasets for rice related to a balanced set of three abiotic stresses (salinity, cold and drought) and three biotic stresses (*Xanthomonas* bacteria, fungal and insect) were obtained from Gene Expression Omnibus (GEO) database of NCBI (http://www.ncbi.nlm.nih.gov) with platform GPL2025, as this platform contains as much as 220 microarray experiments (series) comprising 3480 samples/subjects of *Oryza sativa* L.as compared to other platforms. Among these 3480 samples, 550 experimental samples related to these six different biotic and abiotic stresses for rice were taken in this study. Further, the raw .CEL files for these gene expression samples are downloaded for GEO database of NCBI. The detail description about the selected Microarray datasets for rice is given in Table S1 (in .xlx file).

***Data preprocessing***

The preprocessing of the gene expression datasets was done to remove noises, including missing and mislabeled probes (Das et al., 2017). Here, the preprocessing of data was conducted by using Bioconductor platform of R (Gentleman et al., 2004). Initially, the raw CEL files of the collected samples were processed using Robust Multichip Average (RMA) algorithm available in *affy* Bioconductor package of R (Gautier et al., 2004; Bolstad et al., 2003). This RMA procedure involves background correction, quantile normalization and summarization by median polish approach. Further, the log2 scale transformed expression data from RMA for the collected experimental samples were used for meta-analysis to remove the outlier samples. After normalization of the data, we used the z-score method, a Location-Scale approach (Lazar et al.

2013) to remove the batch effects of the genes. Here, the main idea behind the use of z-score method is to transform the data from each batch to have similar (equal) z-score (i.e. function of mean and variance) for each gene. It is assumed that these transformations, while trivially making data more comparable, do not remove any biological signal of interest

***Meta-analysis of gene expression datasets***

Meta-analysis was performed on the collected gene expression samples to remove the unusual or outlier samples from the data. It was performed individually for each stress. Through this, the gene expression samples with mean ≥ $\mu_0$ and standard deviation ≤ $\sigma_0$ are retained and other samples, which do not satisfy this condition are considered as outliers and removed. Further, we validated the homogeneity of the selected samples through correlation analysis, as they were generated over varying experimental conditions. The parameters for meta-analysis in each stress condition are given in Table S2.

**Table S1**. Description about the gene expression studies used in this study (.xlxs) (given separately).

**Table S2**. Parameters for gene expression samples selection.

| Sl. No. | Stress condition | Mean ($\mu_0$) | Standard deviation ($\sigma_0$) | Samples selected |
|---|---|---|---|---|
| 1 | Cold stress | 6.5 | 2.7 | 28 |
| 2 | Drought stress | 6.2 | 2.6 | 70 |
| 3 | Fungal stress | 6.05 | 2.8 | 26 |
| 4 | Insect stress | 7.4 | 2.4 | 18 |

In meta-analysis, the GE samples with mean ($\mu$) ≥ $\mu_0$ and standard deviation ($\sigma$) ≤ $\sigma_0$ were retained for further study and other samples, which do not satisfy this condition were considered as outliers. The values of $\mu_0$ and $\sigma_0$ were chosen in such a way that the uniformity in color of the correlation plot (among samples) is observed at these parameters setting. For cold

stress, the micro-array samples with $\mu \geq 6.5$ and $\sigma \leq 2.7$ were selected. For drought stress, the micro-array samples with $\mu \geq 6.2$ and $\sigma \leq 2.6$ were selected. For fungal stress, the micro-array samples with $\mu \geq 6.05$ and $\sigma \leq 2.8$ were selected. For insect stress, the microarray samples with $\mu \geq 7.4$ and $\sigma \leq 2.4$ were retained for further analysis. At these parameters' settings, the homogeneity of correlation plot is observed for each of the stresses (Figure S1). In other words, the selected GE samples are observed to be highly homogeneous at these parameters setting, though they were generated over varying experimental conditions. Through the above procedure, the GE samples were retained for further study for all the stresses. The detail description about the selected samples is given in Table S1.

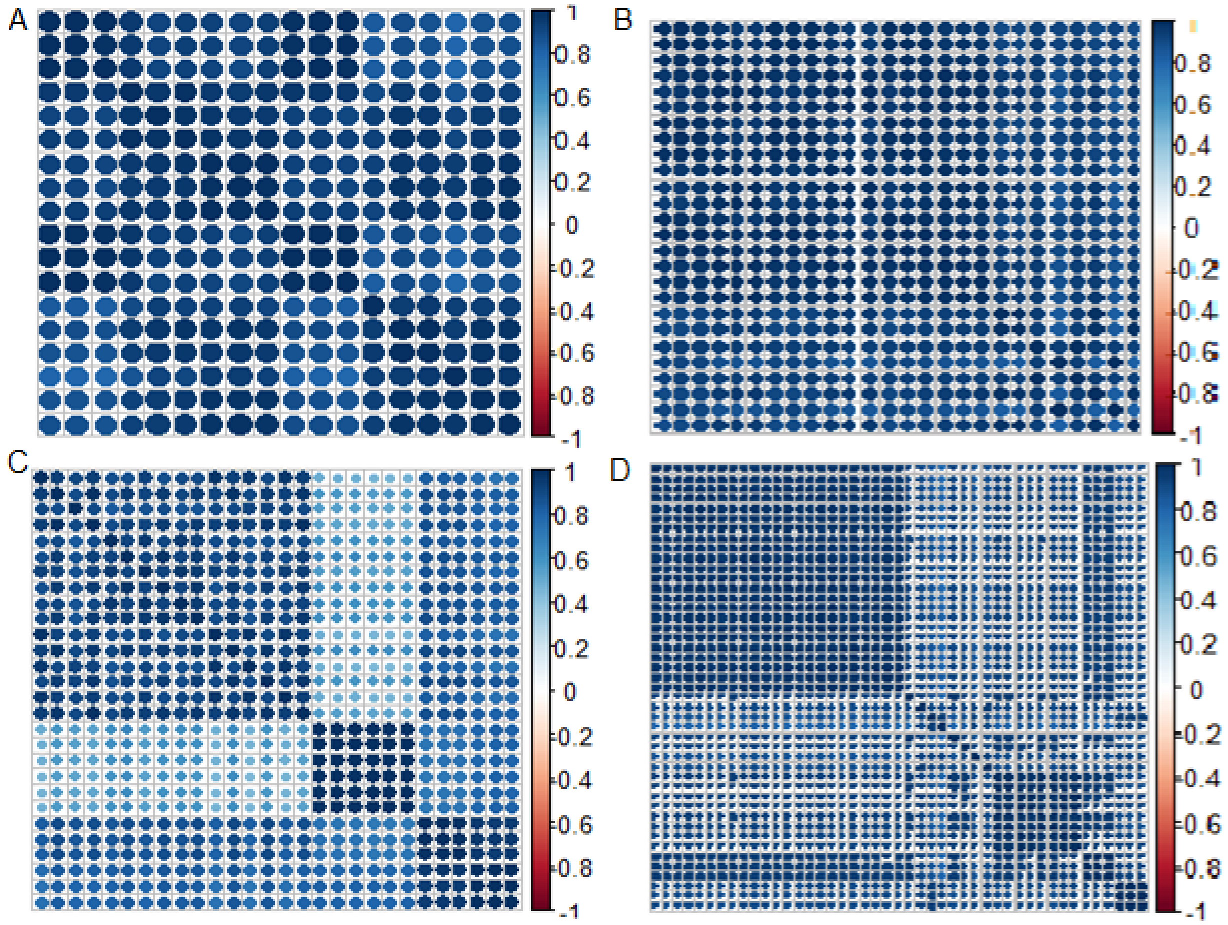


**Figure S1**. Correlation plot of the micro-array experimental samples. The plots are shown for (A) Cold; (B) Drought; (C) Fungal and (D) Insect stresses in rice

Through this meta-analysis, samples which satisfy the above condition are selected for each stress. These parameter combinations are chosen for each stress, as we observed uniformity of color in the correlation plot at these parameter settings, though they are generated over varying experimental conditions across the globe. For instance, in salinity stress, experimental samples whose mean ($\mu$) $\geq$ 5.23 and standard deviation $\leq$ 2.51 were retained for further analysis as they are observed to be highly homogeneous at these parameters setting, irrespective of their

experimental conditions (Figure S1). Similar interpretations can be made for cold, drought, fungal and insect stresses in rice.

***Preliminary gene selection for dimension reduction***

The gene expression data generated from Affymetrix Rice Genome Array (GPL 2025 in GEO), which contains 57,381 probes and each probe is assumed to represent an individual gene. Further, there are 123 probe sets designed for control (in GPL 2025), so, we removed these probes from the analysis and the dataset on 57,258 valid probe sets were obtained for further analysis. It would be of high computational complex as well as statistically infeasible to use the gene set selection methods directly on expression data on 57,258 probes. Hence, we first employed t-test and Fold Change (FC) criteria to filter out unlikely genes to reduce the dimension of the GE datasets at the preliminary stage. In our preliminary selection, we assigned 1 and 0.05 as the |FC| and *p-value* thresholds respectively, resulting in selection of several thousands of genes (Table 1). Further, GE data on these selected genes (at the preliminary stage) were further used for performance analysis of proposed and existing gene selection techniques.

**References**

Das S, Meher PK, Rai A, Bhar LM, Mandal BN. Statistical approaches for gene selection, hub gene identification and module interaction in gene co-expression network analysis: An application to Aluminum stress in Soybean (*Glycine max* L.). PLoS ONE. 2017, 12(1): e0169605. doi:10.1371/journal.pone.0169605.

Gentleman RC, Carey VJ, Bates DM, Bolstad B, Dettling M, Dudoit S, Ellis B, Gautier L, Ge Y, Gentry J (2004) Bioconductor: open software development for computational biology and bioinformatics. Genome Biol 5: 80.

Gautier L, Cope L, Bolstad BM, Irizarry RA (2004) Affy—analysis of Affymetrix GeneChip data at the probe level. Bioinformatics 20: 307–315.

Irizarry RA, Hobbs B, Collin F, Beazer-Barclay,YD, Antonellis KJ, Scherf U, Speed TP (2003) Exploration, normalization, and summaries of high density oligonucleotide array probe level data. Biostatistics 4: 249–264.

Bolstad B, Irizarry R, Astrand M, Speed T (2003) A comparison of normalization methods for high density oligonucleotide array data based on bias and variance. Bioinformatics 19(2): 185–193.

**<u>Document S2:</u> Rice RNA-sequencing dataset collection and pre-processing**

***Raw reads data***

The raw read sequence datasets for the 24 samples/libraries (in. fastq format) were downloaded from the Sequence Read Achieve (SRA) database. The brief description about the collected dataset is given in Table S3. Here, the leaf and root tissue samples were collected from two rice cultivars, i.e., Vialone Nano and Baldo, and the RNA-sequencing (RNA-seq) datasets were generated under two contrasting conditions, salinity treated vs. control. For further statistical analysis, first these raw datasets need to be quality checked, pre-processed, and converted to discrete read counts data. The detailed procedures are discussed in the following sections.

**Table S3**. Description of the RNA-seq data for salinity stress in rice.

| GSM id | SRA id | Samples | Genotype | Tissue | Class | Class label |
|---|---|---|---|---|---|---|
| GSM2940029 | SRR6502085 | SRR6502085_1.fastq | Vialone Nano | Leaves | Salinity | 1 |
| GSM2940030 | SRR6502086 | SRR6502086_1.fastq | Vialone Nano | Leaves | Salinity | 1 |
| GSM2940031 | SRR6502087 | SRR6502087_1.fastq | Vialone Nano | Leaves | Salinity | 1 |
| GSM2940032 | SRR6502088 | SRR6502088_1.fastq | Vialone Nano | Leaves | Control | 0 |
| GSM2940033 | SRR6502089 | SRR6502089_1.fastq | Vialone Nano | Leaves | Control | 0 |
| GSM2940034 | SRR6502090 | SRR6502090_1.fastq | Vialone Nano | Leaves | Control | 0 |
| GSM2940035 | SRR6502091 | SRR6502091_1.fastq | Vialone Nano | Roots | Salinity | 1 |
| GSM2940036 | SRR6502092 | SRR6502092_1.fastq | Vialone Nano | Roots | Salinity | 1 |
| GSM2940037 | SRR6502093 | SRR6502093_1.fastq | Vialone Nano | Roots | Salinity | 1 |
| GSM2940038 | SRR6502094 | SRR6502094_1.fastq | Vialone Nano | Roots | Control | 0 |
| GSM2940039 | SRR6502095 | SRR6502095_1.fastq | Vialone Nano | Roots | Control | 0 |
| GSM2940040 | SRR6502096 | SRR6502096_1.fastq | Vialone Nano | Roots | Control | 0 |
| GSM2940041 | SRR6502097 | SRR6502097_1.fastq | Baldo | Leaves | Salinity | 1 |
| GSM2940042 | SRR6502098 | SRR6502098_1.fastq | Baldo | Leaves | Salinity | 1 |
| GSM2940043 | SRR6502099 | SRR6502099_1.fastq | Baldo | Leaves | Salinity | 1 |
| GSM2940044 | SRR6502100 | SRR6502100_1.fastq | Baldo | Leaves | Control | 0 |
| GSM2940045 | SRR6502101 | SRR6502101_1.fastq | Baldo | Leaves | Control | 0 |
| GSM2940046 | SRR6502102 | SRR6502102_1.fastq | Baldo | Leaves | Control | 0 |
| GSM2940047 | SRR6502103 | SRR6502103_1.fastq | Baldo | Roots | Salinity | 1 |
| GSM2940048 | SRR6502104 | SRR6502104_1.fastq | Baldo | Roots | Salinity | 1 |
| GSM2940049 | SRR6502105 | SRR6502105_1.fastq | Baldo | Roots | Salinity | 1 |
| GSM2940050 | SRR6502106 | SRR6502106_1.fastq | Baldo | Roots | Control | 0 |
| GSM2940051 | SRR6502107 | SRR6502107_1.fastq | Baldo | Roots | Control | 0 |
| GSM2940052 | SRR6502108 | SRR6502108_1.fastq | Baldo | Roots | Control | 0 |

## *Quality check of raw reads*

One of the critical challenges in the RNA-seq study is to ensure that the quality of the samples is maintained properly without contamination. The inclusion of contaminated samples will bias the results of the data analysis. Low-quality libraries in RNA-seq data can stem from damaged or stressed tissues or errors in library preparation. FastQC[1] is one of the most commonly used quality control tools developed for RNA-seq. The output from FastQC is an HTML file viewed in a browser after analyzing a read file in FASTQ format. It gives quality statistics from sequencing data for RNA-seq data and contains information about the input FASTQ file, type of quality score encoding, the total number of reads, read length, and GC content. The first plot*Per base sequence quality*, gives quality score distribution over all the sequenceson the X-axis and the observed mean quality on the Y-axis. The red line in the center of the box and whisker plot gives the median value, the yellow box of the plot represents the inter-quartile range (25-75%), the upper and lower whiskers of the plot represent the 10% and 90% percentile scores, and the blue line represents the mean quality of the read.

The sequence qualities are stored in the form of a Phred score. The Phred score is an error probability belonging to each base and is calculated by:

$$Q = -10 \log_{10} P \tag{1}$$

where Q is the quality score,and P is the error rate. For instance, if Q=30 is assigned to a base, this means the chances that a base is called incorrectly is 1 in 1000($p=10^{-3}$; error rate is 0.1%).A high-quality score implies that a base call is more reliable and less likely to be incorrect; for example, $p=10^{-10}$, Q=100 (1 in 10 billion), which is unrealistic and unlikely. The background of the plotis colored and divides the Y-axis into excellent quality base calls (green), base calls of reasonable quality (orange), and base calls of low quality (red). The median quality score is low

for the first few bases and then rises to gradually drop towards the end of the read, indicating good quality read. A warning is flagged if the median quality for any base is less than 27, while the failure occurs if the median quality falls below 20. The nextplot*Per sequence quality score*, gives the distribution of the average quality score. This quality report allows a check if a subset of sequences has low-quality values universally. There is a problem with the run if a significant proportion of the sequences in a run have overall low quality. If the observed mean quality is below 27, which equals a 0.2% error rate, a warning is issued. A failure is flagged when the mean quality falls below 20.

In the quality analysis, the *Sequence GC content*, that gives the number of reads vs. GC percentage per read. The distribution of mean GC content may vary to a greater or less extent among transcripts. It can lead to the observed plot to be broader or narrower than the ideal plot of normal distribution. The *Per base N content* gives the percentage of bases at each position with or without a base call. If the curve rises above zero at any place, it indicates a problem in the sequencing run. The *Sequence length distribution* plot shows the distribution of fragment sizes in the file. The graph usually has one peak depicting the length of the read but flags a warning if multiple fragment lengths are there in the file. The *Sequence duplication plot* gives the percentage of reads of any given sequence in the file, which occurs several times in the file. For a high coverage library, the exact same region should not be sequenced multiple times. There are two lines in the plot. The blue line gives the distribution of duplication in all the reads, and the red contains duplication levels in the de-duplicated sequences. The *Overrepresented sequence* lists the sequence which appears more than expected in the file. For RNA-Seq data, it is usual that few transcripts may be counted as overrepresented sequences due to the high abundance. A sequence is considered overrepresented if it is accounted for ≥ 0.1% of the total reads for which a

warning is raised and a failure if it is >1%. The *Adapter content* plots the fraction of reads where the sequence library adapter sequence is identified. Through Trimmomatic tool, we removed the adopter sequences from the raw sequence reads data and assessed the quality of the read sequences data using FastQC. The results are shown in Figure S2 – S4.

**Figure S2. Quality plot for the processed raw sequence reads data (part I)**. The quality scores of the base pairs are plotted with base pairs at each position. The plots are shown for (A) SRR6502085; (B) SRR6502086; (C) SRR6502087; (D) SRR6502088; (E) SRR6502089; (F) SRR6502090; (G) SRR6502091; (H) SRR6502092.

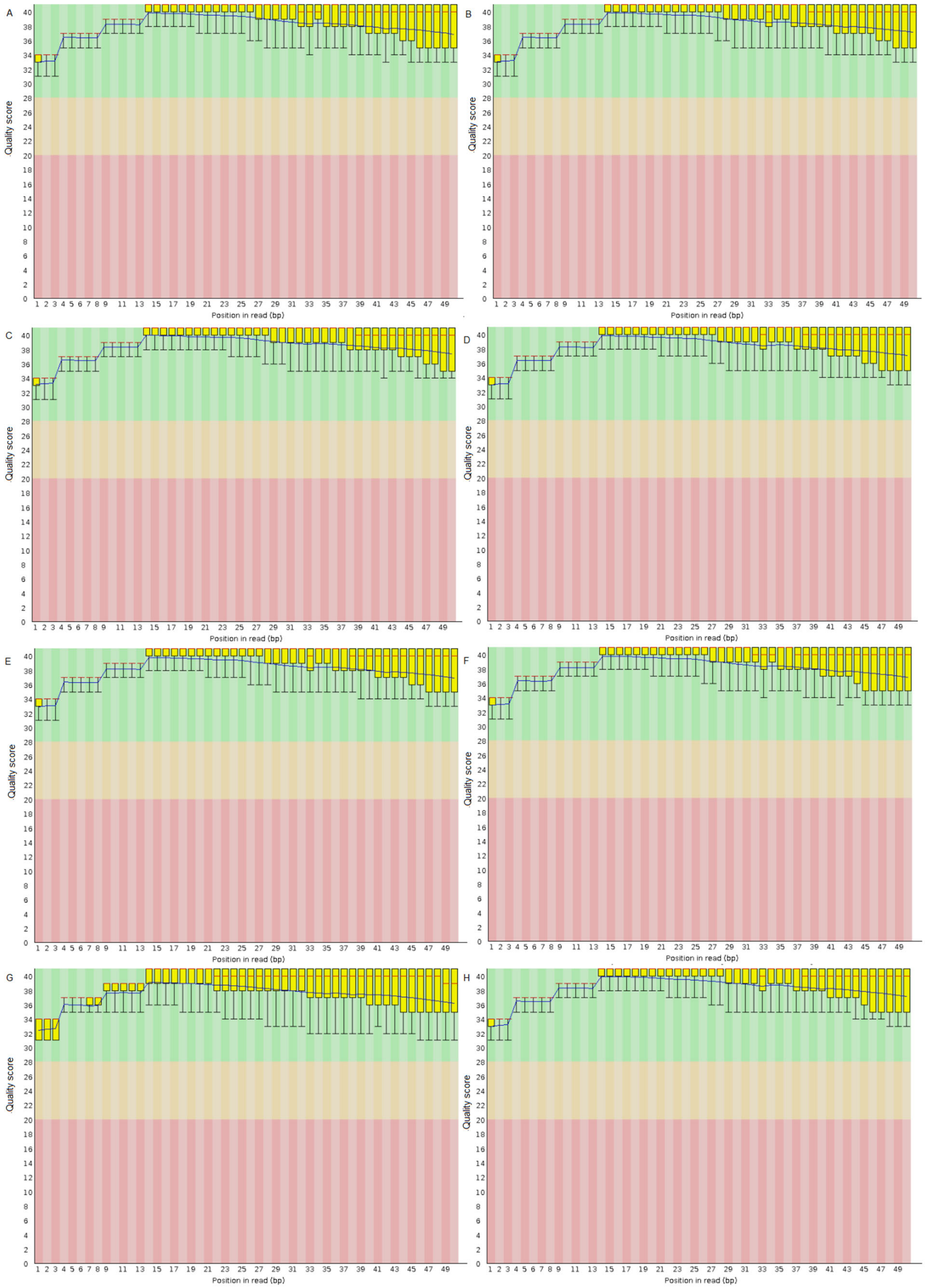
A
B
C
D
E
F
G
H
Quality score
Position in read (bp)

**Figure S3**. Quality plot for the processed raw sequence reads data (part II). The quality scores of the base pairs are plotted with base pairs at each position. The plots are shown for

(A) SRR6502093

(B) SRR6502094

(C) SRR6502095

(D) SRR6502096

(E) SRR6502097

(F) SRR6502098

(G) SRR6502099

(H) SRR6502100

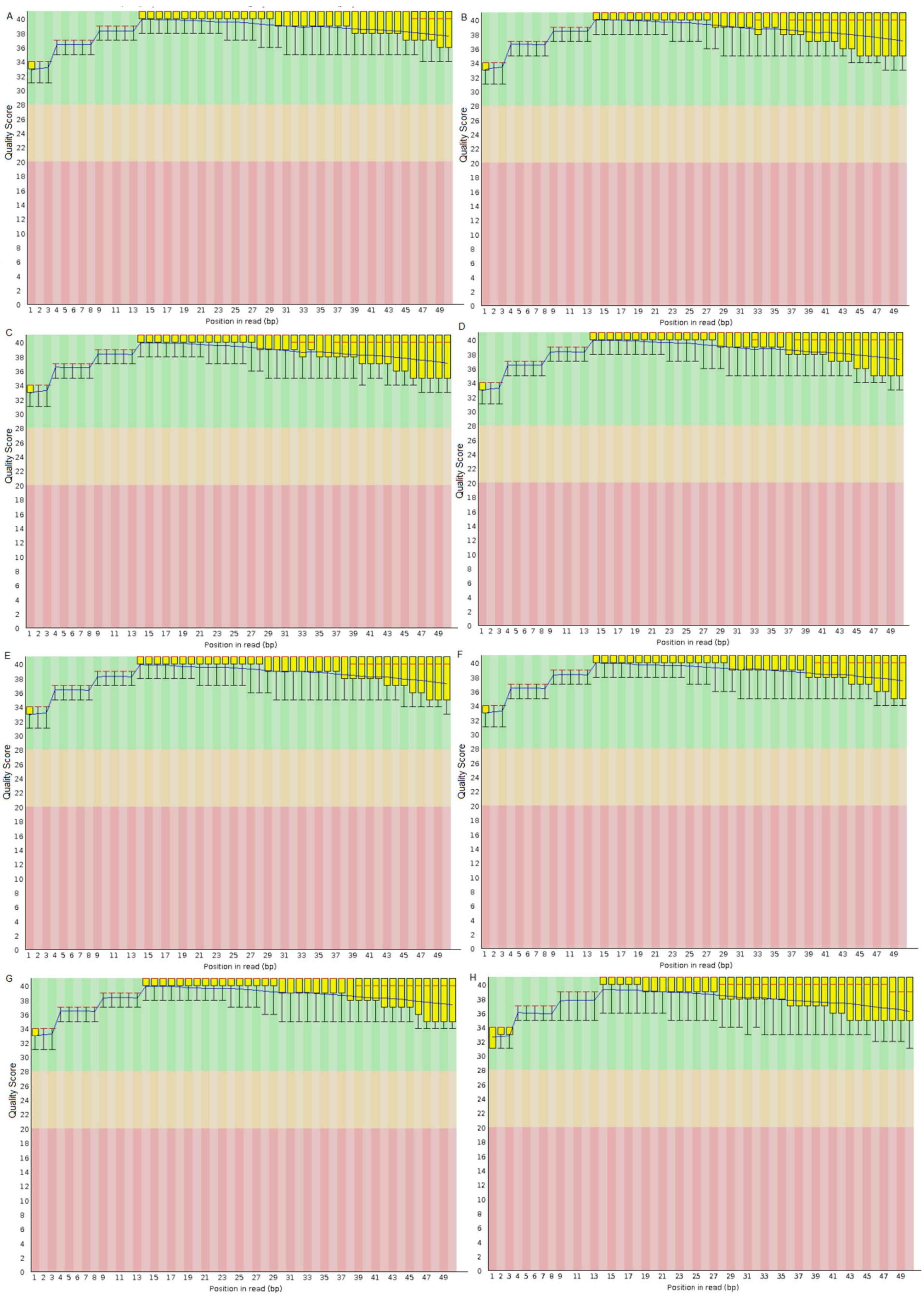
A
B
C
D
E
F
G
H
Quality Score
Position in read (bp)

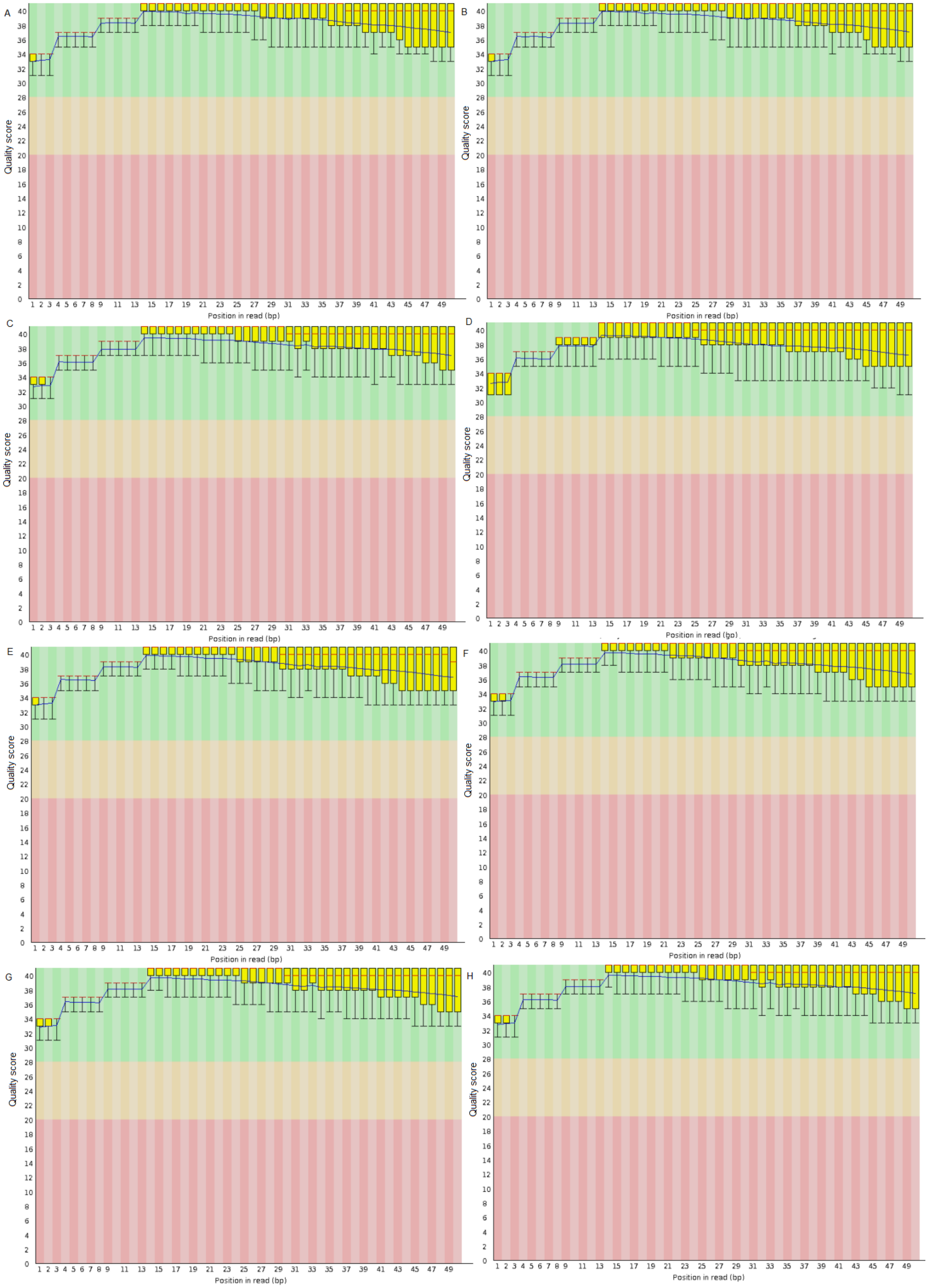
A
B
C
D
E
F
G
H
Quality score
Position in read (bp)

**Figure S4**. Quality plot for the processed raw sequence reads data (part III). The quality scores of the base pairs are plotted with base pairs at each position. The plots are shown for

(A) SRR6502101

(B) SRR6502102

(C) SRR6502103

(D) SRR6502104

(E) SRR6502105

(F) SRR6502106

(G) SRR6502107

(H) SRR6502108

**Document S3. Differential Expression analysis of RNA-seq data**

Usually the Differential Expression (DE) analysis was performed, after mapping the sequence reads to the reference genome followed by quantifying the transcripts, to identify the DE genes (DEG) with respect to two differential conditions (i.e. salinity vs. control). For identification of DEGs, the raw counts were used as input of a state-of-the-art differential expression analysis workflow (Anders et al., 2013). For this purpose, the *edgeR* (v. 3.8.6) bioconductor package implemented on the R language was executed (Robinson et al., 2010). Different sets of DEGs were obtained by comparing RNA-seq libraries/samples under two differential conditions (i.e. treated (salinity) vs. control) for both two tissue samples (i.e. leaves and roots) separately for both cultivars (i.e. Baldo and Vialone Nano). For each comparison, reads with above 1 count per million (CPM) in 3 samples were included in the Generalized Linear Model (GLM) based-pipeline. The normalization factor and the estimated dispersion were computed, both "trended" (or, whenever not possible, "common") and "tagwise," before fitting a GLM to each gene.

Finally, likelihood ratio test was performed for each gene separately and statistical significance values (p-values) was computed for each. Further, the adjusted p-values were computed through Benjamini and Hochberg correction for multiple testing (Benjamini and Hochberg, 1995). The results from the DE analysis is shown in Figure S3.

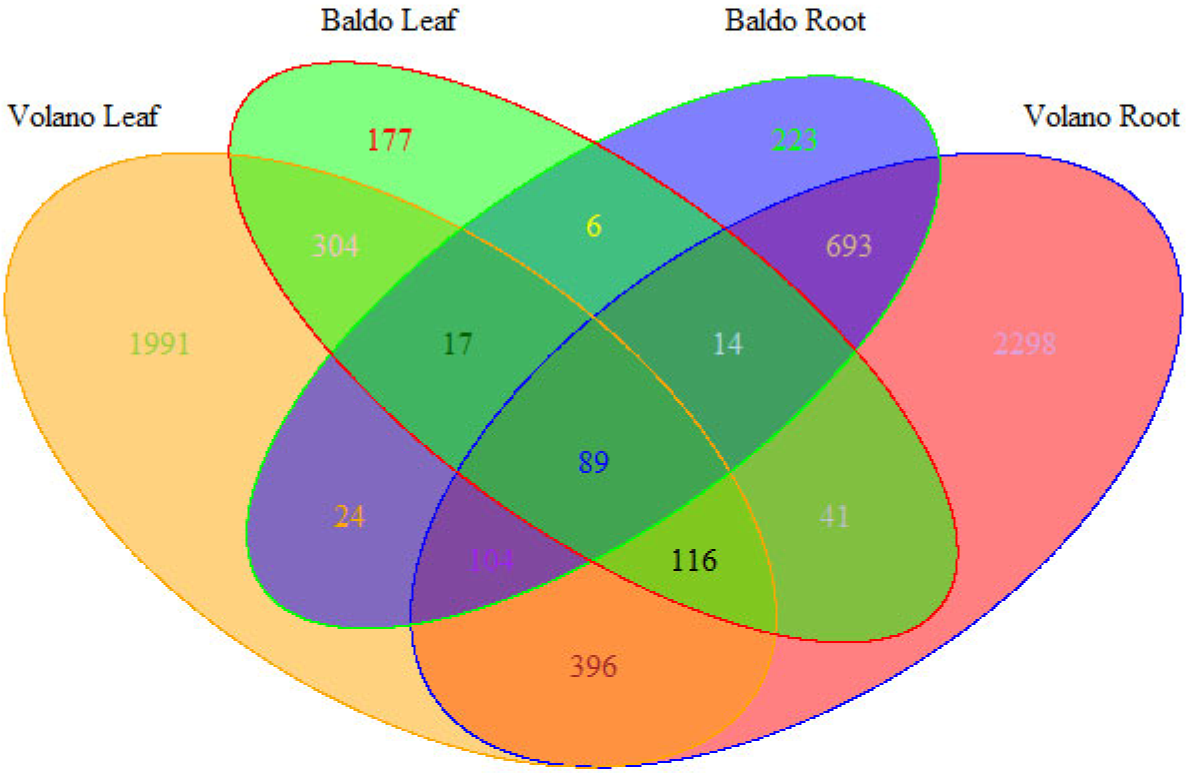


**Figure S5. The DE analysis result and the common DE genes detected between tissue types and genotypes.**

Further, the processed sequence count data was used for differential expression analysis for each tissue type and cultivars separately. The adjusted p-values were computed by using Benjamini and Hochberg correction for multiple hypothesis testing through likelihood ratio test implemented edgeR package of R. The DEGs were identified whose p-values are less than 0.001.

Through this, 3750 and 3041 genes are identified as differentially expressed in root and leaf tissue samples for the Vialone Nano cultivar. Similarly, 1170 and 764 are as DEGs for root and leaf tissue samples of the Baldo cultivar (Figure S5). From these results, it was evident that large number of genes are identified as DEGs in root tissue samples as compared to leaves, due to their direct exposure to salt ions in soil and water for both the cultivars. Moreover, large number of genes are identified as DEGs for the Vialone Nano cultivar as compared to Baldo, which indicated the salinity tolerant characteristics of Vialone Nano.

**Document S4: Stress(s) specific Quantitative Trait Loci information for rice (*Oryza sativa L.*)**

The list of trait specific Quantitative Trait Loci (QTL) for the abiotic, *viz.* salinity, drought, and cold, and biotic stresses bacteria (*Xanthomonas*), fungal (blast) and insect (brown plant hopper) for rice were collected from the Gramene QTL database (http://www.gramene.org/qtl/) (Ni et al. 2009). Then, the genomic regions of these QTLs (for each stress) were mapped to rice genome using Gramene annotation of rice genome of MSU Rice Genome Annotation (Osa1) Release 6 (Ouyang et al. 2007). For a given QTL, there may be 25–30 genes per cM (~270 kbp in rice) (Khurana and Gaikwad, 2005). Further, the lists of the QTLs for each of the six different stresses are given in Table S2-S6.

**Table S4**. List of salinity responsive QTLs in rice (*Oryza sativa L.*).

| Sl. No. | QTL ID | Chr. No. | Start | End | Note |
|---|---|---|---|---|---|
| 1 | AQEM001 | 1 | 33956950 | 37713775 | |
| 2 | AQEM006 | 1 | 9820009 | 11232822 | |
| 3 | AQGR001 | 1 | 38530957 | 38531467 | |
| 4 | AQGR002 | 3 | 22798284 | 22830744 | |
| 5 | AQCL001 | 3 | 484860 | 485333 | |
| 6 | AQEM009 | 4 | 19928370 | 22355854 | |
| 7 | AQCL002 | 4 | 33663984 | 33664487 | |
| 8 | AQCL003 | 5 | 18874932 | 18875558 | |
| 9 | AQCL004 | 6 | 22862400 | 22862821 | |
| 10 | AQEM002 | 6 | 21605889 | 24919236 | |

| | | | | | |
|---|---|---|---|---|---|
| 11 | AQEM003 | 7 | 4573316 | 7739951 | |
| 12 | AQEM004 | 7 | 2633784 | 4575215 | |
| 13 | AQEM007 | 9 | 14362062 | 17837010 | |
| 14 | AQEM005 | 1 | 33956950 | 37713775 | Same as 1 |
| 15 | AQEM008 | 7 | 2633784 | 4575215 | Same as 12 |
| 16 | AQEM010 | 7 | 2633784 | 4575215 | Same as 12 |
| 17 | AQEM011 | 7 | 2633784 | 4575215 | Same as 12 |

Sl. No.: Serial number of the QTL; QTL ID: Published qtl id; Chr. No.: Chromosome number of the QTL; Start: start position of the QTL in terms of base pairs (bp); End: end position of the QTL in terms of length of bps.

**Table S5**. List of cold responsive QTLs for rice (*Oryza sativa L.*)

| Sl. No. | QTL ID | Chr. No. | Start | End | Note |
|---|---|---|---|---|---|
| 1 | CQAA8 | 4 | 688353 | 6574518 | |
| 2 | AQDU008 | 4 | 688353 | 6574518 | Same as 1 |
| 3 | AQDU004 | 11 | 932068 | 932221 | |
| 4 | CQP8 | 11 | 1491600 | 2523808 | |
| 5 | AQDU009 | 6 | 5425408 | 5425631 | |
| 6 | CQAA9 | 6 | 5425408 | 5425631 | Same as 5 |
| 7 | AQAV003 | 1 | 5558576 | 7445919 | |
| 8 | AQDU014 | 12 | 8826555 | 8826855 | |
| 9 | AQDU005 | 12 | 8826555 | 8826855 | Same as 8 |
| 10 | AQAV004 | 2 | 11389704 | 12216613 | |
| 11 | AQAV001 | 2 | 11389704 | 12216613 | Same as 10 |
| 12 | AQAV002 | 9 | 17719660 | 18810331 | |
| 13 | AQF129 | 8 | 19051713 | 22886866 | |
| 14 | AQDU002 | 6 | 19499320 | 27252383 | |
| 15 | AQDU003 | 8 | 20650060 | 21142502 | |
| 16 | AQDU012 | 8 | 20650060 | 21142502 | Same as 15 |
| 17 | AQDU010 | 8 | 21142348 | 21142502 | Same as 15 |
| 18 | CQAA10 | 8 | 21142348 | 21142502 | Same as 15 |
| 19 | CQP7 | 7 | 22857717 | 22885543 | |
| 20 | AQDU013 | 11 | 25153466 | 25153681 | |
| 21 | AQDU015 | 4 | 26857374 | 29061127 | |
| 22 | AQBO001 | 7 | 27159051 | 27159261 | |
| 23 | AQAV006 | 5 | 27342022 | 27342124 | |
| 24 | CQO3 | 4 | 29155838 | 30445683 | |
| 25 | AQDU001 | 4 | 30772388 | 32650528 | |
| 26 | CQO1 | 4 | 31276528 | 32772351 | |
| 27 | CQAA6 | 1 | 32099566 | 33677892 | |
| 28 | AQDU006 | 1 | 32099566 | 33677892 | Same as 27 |
| 29 | CQP1 | 1 | 34651088 | 39949610 | |

Sl. No.: Serial number of the QTL; QTL ID: Published qtl id; Chr. No.: Chromosome number of the QTL; Start: start position of the QTL in terms of base pairs (bp); End: end position of the QTL in terms of length of bps.

**Table S6**: List of drought responsive QTLs in rice (*Oryza sativa L.*)

| Sl. No. | QTL ID | Chr. | Start | End | Note |
|---|---|---|---|---|---|
| 1 | CQAI48 | 4 | 13634515 | 13635012 | |
| 2 | AQA046 | 12 | 26017140 | 27489485 | |
| 3 | AQA045 | 4 | 13634515 | 13635012 | Same as 1 |
| 4 | CQAI49 | 12 | 26017140 | 27489485 | Same as 2 |
| 5 | AQHP062 | 8 | 21645663 | 21647445 | |
| 6 | AQHP058 | 2 | 10503368 | 10503846 | |
| 7 | AQHP059 | 4 | 31662839 | 31663326 | |
| 8 | AQHP082 | 6 | 6718648 | 9537772 | |
| 9 | AQHP083 | 7 | 13074864 | 13075056 | |
| 10 | AQHP070 | 4 | 31662839 | 31663326 | Same as 7 |
| 11 | AQHP065 | 1 | 29184260 | 29184844 | |
| 12 | AQHP081 | 4 | 8610617 | 8611256 | |
| 13 | AQHP079 | 3 | 15469002 | 19412007 | |
| 14 | AQAN005 | 8 | 20094533 | 20094695 | |
| 15 | AQAN004 | 4 | 8610617 | 8611256 | Same as 12 |
| 16 | AQHP066 | 2 | 29761981 | 29762453 | |
| 17 | AQHP080 | 4 | 31662839 | 31663326 | Same as 7 |
| 18 | AQHP084 | 8 | 21645663 | 21647445 | Same as 5 |
| 19 | AQHP069 | 3 | 22798284 | 35828040 | |
| 20 | AQHP068 | 2 | 10503368 | 19866086 | |
| 21 | AQAN001 | 5 | 27342022 | 28610866 | |
| 22 | AQHP078 | 11 | 4413928 | 4415836 | |
| 23 | AQHP067 | 2 | 27034342 | 27035328 | |
| 24 | AQHP085 | 9 | 20481606 | 20482133 | |
| 25 | AQHP061 | 6 | 2560318 | 2561213 | |
| 26 | AQHP075 | 9 | 20481606 | 20482133 | Same as 24 |
| 27 | AQHP060 | 4 | 8610617 | 8611256 | Same as 12 |
| 28 | AQHP074 | 8 | 21645663 | 21647445 | Same as 5 |
| 29 | AQHP087 | 11 | 19565059 | 19565672 | |

Sl. No.: Serial number of the QTL; QTL ID: Published qtl id; Chr.: Chromosome number of the QTL; Start: start position of the QTL in terms of base pairs (bp); End: end position of the QTL in terms of length of bps.

**Table S7.** List of blast (fungal) responsive unique QTLs in rice (*Oryza sativa L.*).

| Sl. No. | QTL ID | Chr. | Start | Stop |
|---|---|---|---|---|
| 1 | AQAF003 | 1 | 1,98,822 | 18,91,260 |
| 2 | AQEN002 | 1 | 50,94,276 | 1,10,77,990 |
| 3 | AQAH002 | 1 | 2,41,86,290 | 2,91,84,844 |
| 4 | AQEN079 | 1 | 50,94,276 | 50,95,699 |
| 5 | AQGJ001 | 1 | 2,94,46,995 | 2,94,47,853 |
| 6 | AQAF011 | 1 | 3,10,46,003 | 3,10,47,458 |
| 7 | AQAF013 | 1 | 3,30,53,493 | 4,00,65,325 |
| 8 | AQAF015 | 1 | 3,44,70,620 | 4,00,65,325 |
| 9 | AQAF017 | 1 | 3,44,70,620 | 3,77,13,775 |
| 10 | AQAQ008 | 1 | 3,07,37,705 | 4,05,67,354 |
| 11 | AQAF006 | 1 | 74,45,627 | 7445919 |
| 12 | AQEN001 | 1 | 1,46,20,467 | 3,49,40,769 |
| 13 | AQAF007 | 1 | 79,70,722 | 79,70,839 |
| 14 | AQCT001 | 1 | 47,38,488 | 3,01,70,285 |
| 15 | AQEN011 | 1 | 1,46,20,467 | 2,17,01,719 |
| 16 | AQEN051 | 1 | 2,05,98,332 | 2,05,99,810 |
| 17 | AQCT002 | 2 | 3,56,61,689 | 3,56,62,199 |
| 18 | AQAQ001 | 2 | 3,56,61,689 | 3,56,62,199 |
| 19 | AQAF026 | 2 | 3,46,52,316 | 3,51,36,068 |
| 20 | AQEN069 | 2 | 2,74,82,581 | 3,11,07,173 |
| 21 | AQEN070 | 2 | 2,74,82,581 | 27,483,257 |
| 22 | AQEN039 | 3 | 4,84,860 | 4,85,333 |
| 23 | AQEN012 | 3 | 4,84,860 | 1,450,227 |
| 24 | AQEN003 | 3 | 4,84,860 | 34,96,275 |
| 25 | AQAQ020 | 3 | 2,51,28,239 | 25,128,864 |
| 26 | AQAF029 | 3 | 2,30,88,332 | 2,45,95,466 |
| 27 | AQGJ003 | 4 | 86,10,617 | 86,11,256 |
| 28 | AQEN063 | 4 | 3,16,62,839 | 3,16,63,326 |
| 29 | AQAQ015 | 4 | 86,10,617 | 1,12,34,543 |
| 30 | CQAC1 | 4 | 1,99,28,370 | 2,23,55,854 |
| 31 | AQEN061 | 4 | 2,00,87,103 | 2,00,87,362 |
| 32 | AQAQ024 | 5 | 2,25,79,390 | 2,25,80,355 |
| 33 | AQCT003 | 5 | 20,91,276 | 2,782,394 |
| 34 | AQEN041 | 6 | 23,63,670 | 23,63,704 |
| 35 | AQEN005 | 6 | 23,63,670 | 67,20,901 |
| 36 | AQAQ021 | 6 | 2,90,27,995 | 3,09,45,628 |
| 37 | AQEN014 | 6 | 95,36,259 | 2,44,55,212 |
| 38 | AQGJ023 | 6 | 25,60,318 | 62,84,636 |
| 39 | AQEN059 | 6 | 95,36,259 | 95,37,772 |
| 40 | AQGJ008 | 6 | 2,67,07,816 | 26,708,549 |
| 41 | AQCT004 | 6 | 62,83,401 | 6,928,661 |
| 42 | AQAH001 | 6 | 69,27,624 | 6,928,661 |
| 43 | AQEN044 | 7 | 2,94,66,368 | 2,94,67,498 |
| 44 | AQEN007 | 7 | 1,30,74,864 | 29,467,498 |
| 45 | AQAF030 | 7 | 15,36,133 | 2,317,976 |
| 46 | AQEN033 | 7 | 15,36,133 | 1,537,879 |
| 47 | AQAF033 | 7 | 2,54,72,688 | 2,65,29,185 |
| 48 | AQGJ010 | 7 | 2,67,04,922 | 29,467,498 |
| 49 | AQAQ016 | 7 | 1,75,25,817 | 18,686,761 |
| 50 | AQAF031 | 7 | 23,16,691 | 7,232,998 |
| 51 | AQGJ026 | 7 | 71,24,042 | 7,124,718 |
| 52 | AQAF035 | 8 | 41,05,519 | 53,27,118 |
| 53 | AQEN015 | 8 | 41,05,519 | 1,74,38,003 |

| | | | | |
|---|---|---|---|---|
| 54 | AQAF034 | 8 | 41,05,519 | 41,06,001 |
| 55 | AQEN037 | 8 | 41,05,519 | 4,106,001 |
| 56 | AQAF038 | 9 | 1,46,48,372 | 20,174,430 |
| 57 | CQAC3 | 9 | 96,29,362 | 10,801,158 |
| 58 | AQAQ022 | 9 | 12,71,123 | 10,801,158 |
| 59 | AQAF040 | 9 | 1,77,19,660 | 18,810,331 |
| 60 | AQAF041 | 9 | 1,99,46,740 | 20,482,185 |
| 61 | AQGJ027 | 9 | 1,88,10,067 | 1,88,10,331 |
| 62 | AQAF042 | 9 | 2,11,89,110 | 22,196,064 |
| 63 | AQCT006 | 10 | 2,09,76,812 | 20,978,165 |
| 64 | AQEN067 | 11 | 2,03,36,572 | 20,337,612 |
| 65 | AQEN016 | 11 | 1,81,78,768 | 20,337,612 |
| 66 | AQAQ017 | 11 | 1,36,71,613 | 28,412,347 |
| 67 | AQEN081 | 11 | 1,81,78,768 | 18,179,510 |
| 68 | AQAO001 | 11 | 1,78,08,335 | 22,816,523 |
| 69 | AQGJ013 | 11 | 66,86,166 | 6,687,145 |
| 70 | AQCT007 | 11 | 46,24,598 | 46,26,888 |
| 71 | AQAQ009 | 12 | 5,32,909 | 1,595,325 |
| 72 | AQEN017 | 12 | 77,29,365 | 23,775,487 |
| 73 | AQEN047 | 12 | 77,29,365 | 7,729,855 |
| 74 | CQAC4 | 12 | 1,10,58,522 | 18,867,702 |
| 75 | AQCT008 | 12 | 77,29,365 | 13,429,507 |
| 76 | AQAQ011 | 12 | 77,29,365 | 77,29,855 |
| 77 | AQEN072 | 12 | 15,94,823 | 15,95,325 |

Sl. No.: Serial number of the unique QTL; QTL ID: Published qtl id; Chr. No.: Chromosome number of the QTL; Start: start position of the QTL in terms of base pairs (bp); End: end position of the QTL in terms of length of bps.

**Table S8**. List of brown plant hopper (insect) responsive unique QTLs in rice (*Oryza sativa L.*).

| Sl. No. | QTL ID | Chr. | Start | Stop |
|---|---|---|---|---|
| 1 | AQAP053 | 2 | 7,44,663 | 7,45,178 |
| 2 | AQAP027 | 6 | 17,64,586 | 18,22,651 |
| 3 | CQAM3 | 1 | 4,29,53,262 | 42,955,596 |
| 4 | AQAP058 | 10 | 53,52,766 | 1,58,02,326 |
| 5 | AQAP009 | 8 | 1,39,27,893 | 2,28,86,866 |
| 6 | AQBA009 | 6 | 67,18,648 | 67,20,901 |
| 7 | AQBA003 | 8 | 2,78,22,512 | 2,78,25,271 |
| 8 | AQBA005 | 4 | 3,36,63,984 | 3,46,98,383 |
| 9 | AQW015 | 12 | 2,74,88,270 | 2,74,89,485 |
| 10 | AQW012 | 11 | 2,31,54,725 | 2,31,55,291 |
| 11 | AQBA002 | 6 | 67,18,648 | 6,720,901 |
| 12 | AQAP023 | 5 | 1,88,74,932 | 22,580,355 |
| 13 | AQAP040 | 6 | 1,70,54,655 | 17,055,184 |
| 14 | AQAP032 | 10 | 2,10,98,188 | 2,10,99,881 |
| 15 | AQAP054 | 3 | 32,36,247 | 3,236,745 |
| 16 | AQAP048 | 4 | 3,06,30,093 | 3,06,30,917 |
| 17 | AQAP036 | 12 | 2,61,07,904 | 26,992,979 |
| 18 | AQAP050 | 11 | 1,36,71,613 | 2,31,55,291 |
| 19 | AQAP051 | 2 | 2,58,65,334 | 27,610,063 |
| 20 | AQAP043 | 5 | 2,25,79,390 | 29,285,656 |
| 21 | AQAP018 | 8 | 2,28,85,196 | 26,282,308 |
| 22 | AQAP035 | 9 | 2,21,94,746 | 2,21,96,064 |
| 23 | AQAP042 | 9 | 2,21,94,746 | 22,196,064 |
| 24 | AQAP029 | 6 | 39,22,784 | 26,708,549 |
| 25 | AQW010 | 6 | 67,18,648 | 19,338,095 |
| 26 | AQBA020 | 6 | 67,18,648 | 1,93,38,095 |
| 27 | AQAP015 | 1 | 2,76,25,475 | 2,91,84,844 |
| 28 | AQAP028 | 8 | 89,23,052 | 8,924,004 |
| 29 | CQAM2 | 6 | 41,60,454 | 8,066,358 |
| 30 | AQW007 | 3 | 57,29,669 | 7,350,653 |
| 31 | AQAP004 | 3 | 3,03,13,472 | 3,03,15,075 |
| 32 | CQT1 | 4 | 35,46,753 | 14,707,274 |
| 33 | AQAP030 | 11 | 1,36,71,613 | 28,412,347 |
| 34 | AQAP045 | 1 | 3,96,87,395 | 4,05,67,354 |
| 35 | AQAP005 | 5 | 52,55,880 | 52,56,140 |
| 36 | AQBA017 | 1 | 1,46,20,467 | 14,626,881 |
| 37 | AQAP056 | 6 | 31,68,314 | 54,25,631 |
| 38 | AQAP057 | 7 | 1,75,25,817 | 2,57,75,868 |
| 39 | AQAP039 | 1 | 10,39,086 | 10,39,868 |
| 40 | AQBA007 | 2 | 1,98,65,083 | 2,45,66,182 |
| 41 | AQAU001 | 2 | 89,84,645 | 18,249,617 |
| 42 | CQT2 | 3 | 3,19,45,962 | 35,710,936 |
| 43 | AQW013 | 12 | 1,96,28,443 | 19,628,925 |
| 44 | AQAP034 | 11 | 57,06,417 | 57,06,935 |
| 45 | AQAP041 | 2 | 45,24,663 | 5,263,536 |
| 46 | AQAP055 | 4 | 2,01,71,917 | 2,01,73,040 |
| 47 | AQW014 | 12 | 1,96,28,443 | 19,628,925 |
| 48 | AQBA022 | 1 | 1,46,20,467 | 14,626,881 |
| 49 | AQW008 | 4 | 2,41,65,104 | 24,165,408 |
| 50 | AQAU003 | 12 | 5,32,909 | 5,33,313 |
| 51 | AQAP007 | 5 | 52,55,880 | 6,700,408 |
| 52 | AQAU002 | 10 | 2,14,02,080 | 23,031,714 |
| 53 | AQAP031 | 8 | 1,39,27,893 | 2,06,50,257 |

| | | | | |
|---|---|---|---|---|
| 54 | AQAP052 | 2 | 52,62,891 | 69,16,662 |
| 55 | AQBA008 | 4 | 3,16,62,839 | 32,449,446 |
| 56 | AQAP046 | 10 | 1,77,94,267 | 1,98,23,295 |
| 57 | AQAP001 | 1 | 97,01,793 | 1,04,91,821 |

Sl. No.: Serial number of the unique QTL; QTL ID: Published qtl id; Chr. No.: Chromosome number of the QTL; Start: start position of the QTL in terms of base pairs (bp); End: end position of the QTL in terms of length of bps.

**Table S9.** Number of gene samples and sizes of gene sample for each selected gene set for GSAQ analysis.

| Sl. No. | Selected gene set size (*n*) | Gene sample size (*m*) | No. of gene samples (*K*) |
|---|---|---|---|
| 1 | 200 | 170 | 50 |
| 2 | 300 | 255 | 50 |
| 3 | 400 | 340 | 60 |
| 4 | 500 | 425 | 60 |
| 5 | 600 | 510 | 60 |
| 6 | 700 | 595 | 60 |
| 7 | 800 | 680 | 70 |
| 8 | 900 | 765 | 70 |
| 9 | 1000 | 850 | 70 |
| 10 | 1100 | 935 | 70 |
| 11 | 1200 | 1020 | 80 |
| 12 | 1300 | 1105 | 80 |
| 13 | 1400 | 1190 | 80 |
| 14 | 1500 | 1275 | 90 |
| 15 | 1600 | 1360 | 90 |
| 16 | 1700 | 1445 | 90 |
| 17 | 1800 | 1530 | 100 |
| 18 | 1900 | 1615 | 100 |
| 19 | 2000 | 1700 | 100 |

## Document S5:Guidelines and tutorials for GSQSeq R package

### Inputs for GSQSeq R package

1. Gene Expression count data/Microarraymatrix ***X*** with *N* genes and *M* samples/libraries
2. Class information *M*-dimensional vector having elements +1 (case) and 0 (control)
3. QTL dataset ***Q*** with rows as *K* QTL ids and columns as chromosome number, start and stop positions of the QTL id.
4. Gene space (list) dataset with *N* genes as rows and columns as chromosome number, start and stop positions of each gene.
5. Gene Selection Method to produce Gene Set *G*. Includes a simple univariate t-test, F-score, MultivariateMRMR or Machine learning Support Vector Machine for Microarray gene expression data. We use only one probe per gene to prevent overestimation of the NQhits and GSQ statistic(s).
6. Differential expression analysis methods to produce Gene Set *G* for RNA-seq count expression data. This includes either DESeq2 or edgeR methods.
7. Size: Size of the selected gene set selected from the expression data.

### R package Installation from local directory:

```
Required e1071 R package
install.packages("…/…path location to the
file…/GSQSeq_0.1.0.tar.gz", repos = NULL, type = "source")
library(e1071)
library(GSAQ)
library(BootMRMR)
library(GSQSeq)

**** Data example
(Download the data from the supplementary files)
####Example Microarray Data
Expdata<- read.table(file="MicroarrayData.txt", header = TRUE,
row.names = 1, sep="\t")
###Class information
class <- c(rep(1, 35), rep(0, 35))
#####Example QTL data
QTLdata<- read.table(file="QTLData.txt", header = TRUE,
row.names = 1, sep="\t")
####Location of the genes of Microarray data
MicGeneLoc<- read.table(file="MicroGeneLocation.txt", header =
TRUE, row.names = 1, sep="\t")
###Example RNA-seq count data
CountData<- read.table(file="RNACountData.txt", header = TRUE,
row.names = 1, sep="\t")
######Location of the genes in RNA-seq data
RNAGeneLoc<- read.table(file="RNAGeneLocation.txt", header =
TRUE, row.names = 1, sep="\t")
class <- c(rep(1, 12), rep(0, 12))
```

**R package Installation steps from web:**

```
#get the devtools
install.packages("devtools") ##install if not
iibrary(devtool)
#install GSQSeq R package from web
install_github("sam-uofl/GSQSeq")
library(GSQSeq)
```

#**Gene set analysis with QTL for Microarray Data**

```
>GSQMicro(ExpData, class, geneLoc, size, QTLData, method)
```

Arguments:

| | |
|---|---|
| `ExpData` | N by M matrix of expression values, rows as genes (N: total number of genes) and columns as samples (M: Total samples). |
| `class` | Vector of 1 and 0 of length M shows the class labels of samples (1: case and 0: control). |
| `geneLoc` | N by 3 dataframe/ matrix (genes/gene ids as row names); where, N represents the number of genes in the whole gene set: first coloumnrepresnting the chromosomal location of genes: second coloumn representing the start position of genes in terms of basepairs: third coloumn representing the end position of genes in terms of basepairs in their respective chromosomes. |
| `size` | size of the selected gene set to be analyzed with the QTL, e.g. 100, 500, ... |
| `QTLData` | Q by 3 dataframe/matrix (qtl names/qtl ids as row names);where, Q represents the number of qtls: first coloumnrepresnting the chromosomal location of qtls: second coloumn representing the start position of qtls in terms of basepairs: third coloumn representing the end position of qtls in terms of basepairs in their respective chromosomes. |
| `method` | A character representing the Differential Expression (DE) analysis method to be used for DE analysis of expression data. It must be either T-test or F-scores or MRMR or SVM. |

**######Example data analysis**

```
>GSAmicro = GSQMicro(ExpData = Expdata, class = class, geneLoc =
MicGeneLoc, size = 1000, QTLData = QTLdata, method = "MRMR")
Summary(GSAmicro)
```

# **Gene set analysis with QTL for RNA-seq Data**

```
>GSQSeq(CountData, class, geneLoc, size, QTLData, method)
```

## Arguments

| | |
|---|---|
| `CountData` | N by M matrix of reads count data (N: Total number of genes; M: Total number of samples), rows represent the genes and columns represent samples/libraries. |
| `class` | M by 1 vector of 0 and 1 representing the class labels of the samples, i.e. 1 for case and 0 for control. |
| `geneLoc` | N by 3 data frame/ matrix (genes/gene ids as row names); where, N represents the number of genes in the whole gene set: first coloumnrepresnting the chromosomal location of genes: second coloumn representing the start position of genes in terms of basepairs: third coloumn representing the end position of genes in terms of basepairs in their |

respective chromosomes.

`size` size of the selected gene set to be analyzed with the QTL, e.g. 100, 500, ...

`QTLData` Q by 3 data frame/matrix (qtl names/qtl ids as row names);where, Q represents the number of qtls: first coloumnrepresnting the chromosomal location of qtls: second coloumn representing the start position of qtls in terms of basepairs: third coloumn representing the end position of qtls in terms of basepairs in their respective chromosomes.

`method` A character representing the Differential Expression (DE) analysis method to be used for DE analysis of RNA-seq data. It must be either DESeq or edgeR.

```
######Example
GSASeq<- GSQSeq(CountData = RNACountData, class = class, geneLoc =
RNAGeneLoc, size = 200, QTLData = QTLdata, method = "edegeR")

summary (GSASeq)
```

## #Finding QTL enriched gene set.

```
>GeneQTL(geneset, genelist, qtl)
```

### Arguments

`geneset` vector of characters representing the names of genes/ gene ids selected from the whole gene list/space by using a gene selection method.

`genelist` N by 3 dataframe/ matrix (genes/gene ids as row names); where, N represents the number of genes in the whole gene set: first coloumnrepresnting the chromosomal location of genes: second coloumn representing the start position of genes in terms of basepairs: third coloumn representing the end position of genes in terms of basepairs in their respective chromosomes.

`qtl` Q by 3 dataframe/matrix (qtl names/qtl ids as row names);where, Q represents the number of qtls: first coloumnrepresnting the chromosomal location of qtls: second coloumn representing the start position of qtls in terms of basepairs: third coloumn representing the end position of qtls in terms of basepairs in their respective chromosomes.

## Other Materials

**Supplementary Material 1. Example datasets for GSQSeq R package.**

**Supplementary Material 2. GSQSeq R package.**
